# Imaging of the Lymphatic Vessels for Surgical Planning: A Systematic Review

**DOI:** 10.1245/s10434-022-12552-7

**Published:** 2022-09-28

**Authors:** Saskia van Heumen, Jonas J. M. Riksen, Wichor M. Bramer, Gijs van Soest, Dalibor Vasilic

**Affiliations:** 1 Department of Plastic and Reconstructive Surgery, Erasmus MC, University Medical Center, Rotterdam, The Netherlands; 2 Department of Cardiology, Erasmus MC, University Medical Center, Rotterdam, The Netherlands; 3 MSc Educational Program Technical Medicine, Leiden University Medical Center, Delft University of Technology and Erasmus MC, University Medical Center, Rotterdam, The Netherlands; 4 Medical Library, Erasmus MC, University Medical Center, Rotterdam, The Netherlands

## Abstract

**Background:**

Secondary lymphedema is a common complication after surgical or radiotherapeutic cancer treatment. (Micro) surgical intervention such as lymphovenous bypass and vascularized lymph node transfer is a possible solution in patients who are refractory to conventional treatment. Adequate imaging is needed to identify functional lymphatic vessels and nearby veins for surgical planning.

**Methods:**

A systematic literature search of the Embase, MEDLINE ALL via Ovid, Web of Science Core Collection and Cochrane CENTRAL Register of Trials databases was conducted in February 2022. Studies reporting on lymphatic vessel detection in healthy subjects or secondary lymphedema of the limbs or head and neck were analyzed.

**Results:**

Overall, 129 lymphatic vessel imaging studies were included, and six imaging modalities were identified. The aim of the studies was diagnosis, severity staging, and/or surgical planning.

**Conclusion:**

Due to its utility in surgical planning, near-infrared fluorescence lymphangiography (NIRF-L) has gained prominence in recent years relative to lymphoscintigraphy, the current gold standard for diagnosis and severity staging. Magnetic resonance lymphography (MRL) gives three-dimensional detailed information on the location of both lymphatic vessels and veins and the extent of fat hypertrophy; however, MRL is less practical for routine presurgical implementation due to its limited availability and high cost. High frequency ultrasound imaging can provide high resolution imaging of lymphatic vessels but is highly operator-dependent and accurate identification of lymphatic vessels is difficult. Finally, photoacoustic imaging (PAI) is a novel technique for visualization of functional lymphatic vessels and veins. More evidence is needed to evaluate the utility of PAI in surgical planning.

**Supplementary Information:**

The online version contains supplementary material available at 10.1245/s10434-022-12552-7.

The lymphatic system fulfils several functions in the body: primarily, it drains interstitial fluid, transports lipids and proteins, and is an important conduit for mediating the immune response.^1,2^ Lymphedema is the accumulation of lymph fluid in the interstitium, causing swelling of the affected area.^3^ Patients experience discomfort, fatigue, diminished strength, and sometimes recurrent cellulitis, leading to compromised functioning and, in grave cases, irreversible fibrosis. Not surprisingly, a severe negative impact on a person’s quality of life is often reported.^4^

The cause can be either an hereditary or congenital condition (primary lymphedema) or a result from damage to the lymphatic system (secondary lymphedema). The latter is far more common and is often caused by cancer treatment. Although treatments have become less invasive over the years,^5^ approximately one in five breast cancer patients will develop lymphedema,^6^ with lymph node dissection, mastectomy, and radiation therapy as risk factors.^5,7,8^

Primary diagnosis is based on clinical presentation and the medical history of a patient. Clinical severity is often assessed using the International Society of Lymphology (ISL) scale^9^ or the Campisi Clinical scale.^10^ Clinical signs are however subjective and are not always accurate.^11^

Early diagnosis and therapy are essential for patient comfort and preventing loss of function.^12^ Initially, complete decongestive therapy (CDT) is deployed for conservative treatment. Lymphovenous bypass (LVB) and vascularized lymph node transfers (VLNTs) are (micro)surgical interventions, gaining momentum as an important treatment alternative.^13–15^ Reductive procedures (excision or liposuction) are sometimes performed in severe cases.^16^ An important aspect of surgical decision making is the detection of functional, non-sclerotic^17^ lymphatic vessels and the presence of a nearby suitable receiving vein.^18,19^ Therefore, preoperative imaging is of great importance to substantiate treatment choice.

This systematic review presents an overview of the existing imaging modalities used for preoperative visualization of the lymphatic vessels in patients with secondary lymphedema of the extremities or head and neck. We describe the most important findings and advantages and disadvantages for each modality and discuss this from the perspective of surgical interventions. This means that, ideally, imaging should detect lymphatic functionality, show its course in three dimensions, and display the venous network that will function as an anastomotic acceptor site.

## Methods

### Search Strategy

A systematic literature search of the Embase, MEDLINE ALL via Ovid, Web of Science Core Collection and Cochrane CENTRAL Register of Trials databases was conducted on 24 February 2022. The search query was developed by an experienced medical information specialist (WMB) and consisted of synonyms and thesaurus terms of four concepts: (1) lymphatic vessel or lymphography; (2) imaging or different imaging modalities (magnetic resonance, scintigraphy, ultrasound, photoacoustic, fluorescence); (3) lymphedema; and (4) head and neck or extremities. For full details of the search queries, see electronic supplementary Table 1. The search results of all databases were imported in EndNote and deduplicated using the method described by Bramer et al.^20^

### Inclusion and Exclusion Criteria

Studies were included if they described imaging of the lymphatic vessels in healthy participants or in patients with secondary lymphedema affecting the upper or lower extremities or the head and neck. Studies including both primary and secondary lymphedema patients were also included; however, if a study only investigated primary lymphedema patients, it was excluded. Only studies that primarily analyzed one or more imaging modalities for visualization of the lymphatic system and that specifically mentioned visualization of the lymphatic vessels were included. Therefore, studies reporting on the lymph nodes only were not included. Studies on intra- or post-surgical imaging were excluded, as were studies involving animals or cadavers, case reports, reviews, conference proceedings, and commentaries. Articles published before 2000 were also excluded because imaging modalities and devices used before this time were considered obsolete and are often not used in clinical practice anymore. Studies available in English and full-text studies were assessed for eligibility.

### Study Selection and Data Extraction

Search results from all databases were collected and duplicates were removed. All titles and abstracts were retrieved and assessed for eligibility. The remaining records were subsequently assessed based on full text. Eligibility was discussed between two reviewers (SvH, JR) and consensus was reached. The following information was extracted from each study: year of publication, author identification, study population, cause of lymphedema, clinical staging, contrast agent administration information (type, dose, injection site, and injection type), and the imaging device used. Furthermore, outcomes regarding imaging quality of the lymphatic vessels or diagnostic performance were also extracted.

## Results

### Studies Included

**Fig. 1:**
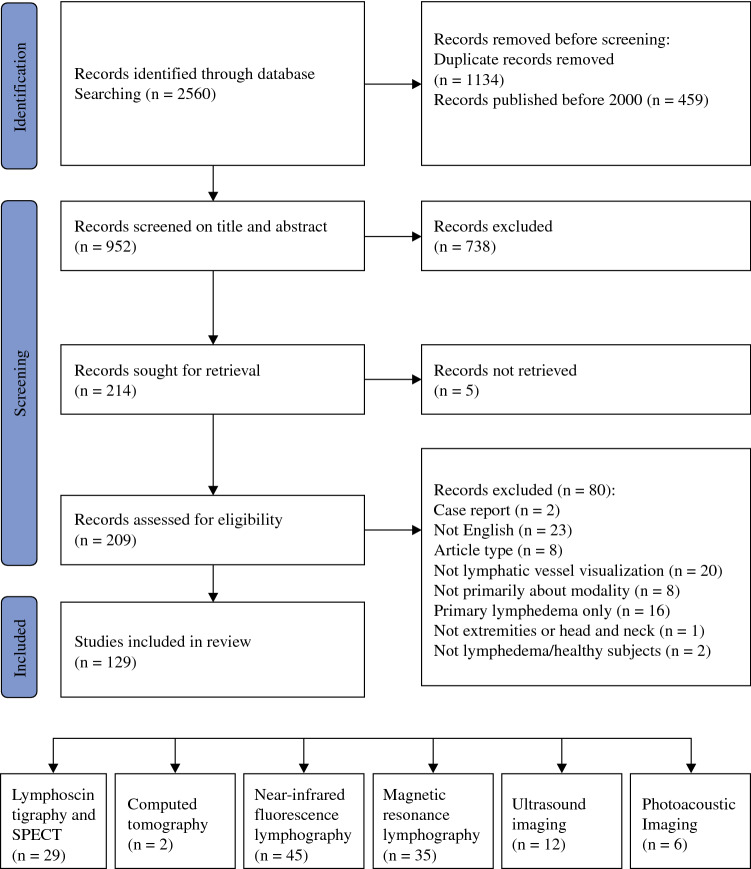
PRISMA flow diagram on study inclusion.

**Fig. 2:**
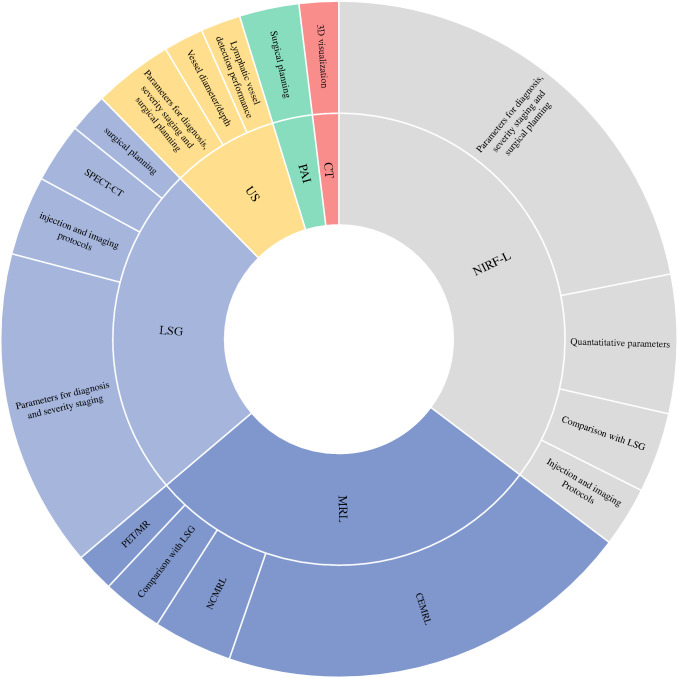
Overview of the included studies and their contribution to the lymphatic vessel imaging field. *LSG* lymphoscintigraphy, *US* ultrasound, *PAI* photoacoustic imaging, *CT* computed tomography, *NIRF-L* near-infrared fluorescence lymphography, *MR* magnetic resonance, *MRL* magnetic resonance lymphography, *CEMRL* contrast-enhanced MRL, *NCMRL* non-contrast MRL, *PET* positron emission tomography, *SPECT* single photon emission computed tomography

After removal of duplicates and studies published before 2000, the literature search resulted in 952 records. Screening resulted in the exclusion of 823 records, leaving 129 records for inclusion. Figure 1 gives an overview of the study inclusion process. Six different imaging modalities were identified, namely lymphoscintigraphy, near-infrared fluorescence lymphangiography (NIRF-L), computed tomography (CT), magnetic resonance lymphography (MRL), ultrasound imaging (US), and photoacoustic imaging (PAI). The included studies had different aims and methods and heterogeneous study populations, and therefore all results are described narratively. Figure 2 gives an overview of the relative contribution of the imaging modalities and the most important subjects discussed.

### Lymphoscintigraphy

**Fig. 3:**
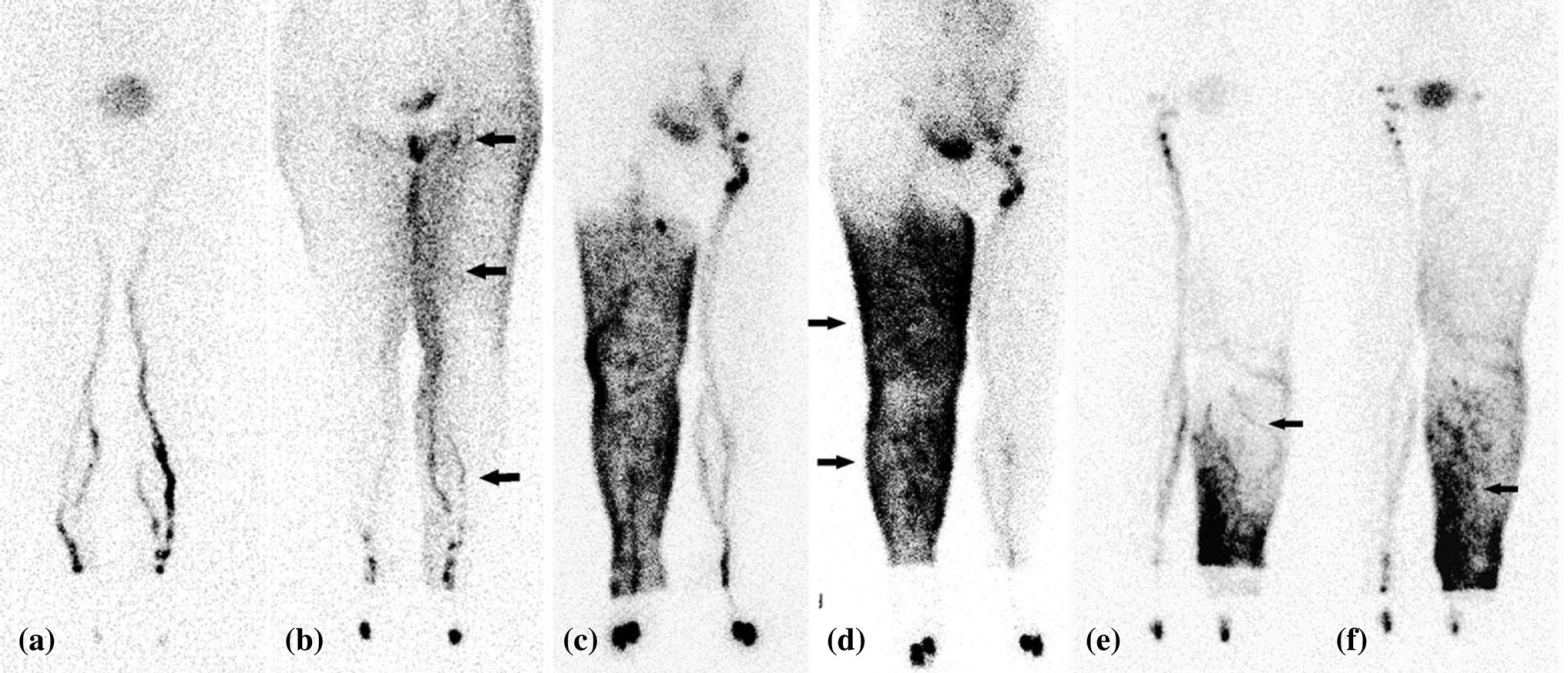
Lymphoscintigraphy imaging of the lymphatics. **a, b** Images of type II in a patient with left lymphedema **a** 30 and **b** 120 min after injection of contrast medium. Lymph stasis in the lymphatics (arrow) and visible dermal backflow (arrow) on the left thigh can be seen. The inguinal lymph nodes are reduced in number (arrow). **c, d** Images of type III in a patient with right lymphedema **c** 30 and **d** 120 min after injection of contrast medium. Dermal backflow (arrows) in the leg and thigh can be seen. **e, f** Images of type IV in a patient with left lymphedema **e** 30 and **f** 120 min after injection of contrast medium. Dermal backflow [arrow in **e**] and lymph stasis in the lymph vessels [arrow in **f**] in the leg can be seen and remains in the leg 120 min later. Reprinted from Maegawa et al.^35^ with permission from John Wiley & Sons, Inc

Of all imaging modalities, lymphoscintigraphy has been used for the longest period of time. With a gamma camera, whole-body images, as shown in Fig. 3, are obtained to get a gross overview of the lymphatic uptake of a 99m-Technetium-labeled contrast agent.^21^ Most studies described methods for lymphedema diagnosis and severity staging, while one study reported on the visualization of head and neck drainage pathways.^22^ Electronic supplementary Table 2 gives an overview of the included lymphoscintigraphy studies.

#### Parameters for Diagnosis and Severity Staging Systems

Several qualitative and quantitative parameters may categorize patients into severity types. There was clear agreement between studies on several factors that contributed to adequate diagnosis and staging, namely visualization of inguinal or axillary lymph nodes, the lymphatic vessels (normal, dilated, or collaterals), lymphatic fluid leakage into the subcutaneous tissue (i.e., dermal backflow [DBF]), and uptake in popliteal or antecubital lymph nodes. Studies evaluated different factors, with variable weighting.

Quantitative scintigraphy parameters reflected the overall functionality of the lymphatics and were primarily derived from the arrival time in the proximal lymph nodes (transit time [TT] or tracer appearance time)^23–28^ or clearance rate from the injection site (depot disappearance rate constant) and subsequent uptake in the blood.^29–33^

The severity of lymphedema was graded using the abovementioned characteristics, and differentiated patients into four,^25,34^ five,^28,35,36^ or six (Taiwan Lymphoscintigraphy Staging)^37,38^ stages. Lymphoscintigraphy findings have been combined with clinical symptoms and circumference measurements in Cheng’s Lymphedema Grade system and earlier scales.^37–39^ Lastly, the transport index^40^ is a scoring of several subjective observations indicating either normal or abnormal lymphatics. Correlations between clinical parameters and lymphoscintigraphy staging systems have been reported^35–38,41^ but were not always significant.^35,36^

#### Diagnostic Performance

Evaluation of both qualitative and quantitative parameters was highly reproducible^32^ but the diagnostic performance differed. High sensitivities (92.3–96%) and specificities (92.9–100%) of lymphedema diagnosis based only on qualitative parameters were reported.^27,42^ On the other hand, a lower sensitivity and specificity of 51% and 89%, respectively, based solely on quantitative parameters, was found, leading to ambiguous diagnoses. Combining quantitative findings with qualitative findings moderately increased the sensitivity, while specificity remained constant.^23^ Qualitative and quantitative scintigraphy parameters correlated variably with limb circumference differences.^26,43^ It was even proposed that lymphoscintigraphy does not give additional information beyond abnormal or normal lymphatics.^26^

#### Treatment Decision Making and Surgical Planning

Different treatment regimens based on a patient’s lymphoscintigraphy stage were proposed.^25,36–38,41^ Overall, CDT was indicated in less severe cases, LVB was indicated in patients with partially obstructed lymphatics,^36^ and VLNT was indicated for patients with severely obstructed lymphatics, sometimes combined with debulking surgery.^37,38^ Differentiation between deep and superficial vessels may be beneficial for treating multiple levels of the lymphatic system.^41^ Other methods were used for intraoperative vessel identification after lymphoscintigraphy-based diagnosis,^35,36^ because its resolution did not permit precise selection of the anastomosis site.

Four studies investigated the predictive value of qualitative lymphoscintigraphy findings and treatment success. No clear relation between lymphatic vessel visualization and CDT treatment success was found;^33^ however, visibly dilated lymph vessels, altered flow, and DBF patterns were significantly related to better LVB surgery outcomes.^44–46^

#### Injection and Imaging Protocols

Contrast agents were generally injected subcutaneously, and intradermal injections in smaller numbers (electronic supplementary Table 3). Intradermal injections yielded better image quality of the superficial lymphatics and are therefore a more accurate assessment. Subsequently, faster uptake of the radiotracer was observed, allowing for shorter imaging durations.^24,28,30,31^ Subfascial tracer injection was not suitable for lymphatic vessel visualization.^29,41^

Imaging protocols (stress vs. rest protocols) differed substantially. The increase in muscle activity in stress-based protocols may facilitate tracer uptake in the lymphatics, which increases the likelihood of successful visualization.^28^ Such protocols may therefore distinguish whether compensatory mechanisms involve the deep or superficial system, which can affect treatment choice.^24^ Lastly, the number and timing of contrast dye injections might influence the findings.^47^

#### Single Photon Emission Computed Tomography

Single photon emission computed tomography (SPECT) combined with CT imaging has widely been used for identification of sentinel lymph nodes but the application for visualizing lymphatic vessels is limited. In contrast to scintigraphy, SPECT/CT provides three-dimensional (3D) and in-depth information,^48,49^ and also provides information on lymphostasis in patients with early lymphedema, which was detected sporadically with planar scintigraphy. SPECT/CT mostly confirmed and improved localized findings from planar scintigraphy, and soft tissue changes could be assessed.^49–51^

### Computed Tomography

One study investigated lopamidol contrast-enhanced CT imaging for lymphatic vessels with the potential benefit of 3D information, relatively high resolution, and short imaging time.^52^ In terms of resolution, CT was better than lymphoscintigraphy but worse than NIRF-L.^52^ Lymphatic vessels were hardly visible above the knee and classification of lymphedema severity based on DBF was not possible.^52^ Moreover, auxiliary information such as presence of fibrosis or fluid retention in the subcutaneous fat did not provide an accurate diagnosis. The diagnostic sensitivity of CT (33%) was inferior to those of NIRF-L (100%), lymphoscintigraphy (66%), or MRI (100%).^53^

### Near-Infrared Fluorescence Lymphangiography (NIRF-L)

NIRF-L uses the fluorescence properties of indocyanine green (ICG)^54^ for real-time visualization of the lymphatic vessels. It is applied to identify normal and altered drainage pathways,^55–60^ evaluating anastomosis patency,^61^ and provides information about vessel functionality by visualizing pulsatile behavior.^59^ It also provides more insight into anatomical variations and the relation between the development of lymphedema after cancer treatment and the formation of accessory pathways.^59,62–64^ Electronic supplementary Table 4 gives an overview of the included NIRF-L studies.

#### Parameters for Diagnosis and Severity Staging Systems

**Fig. 4:**
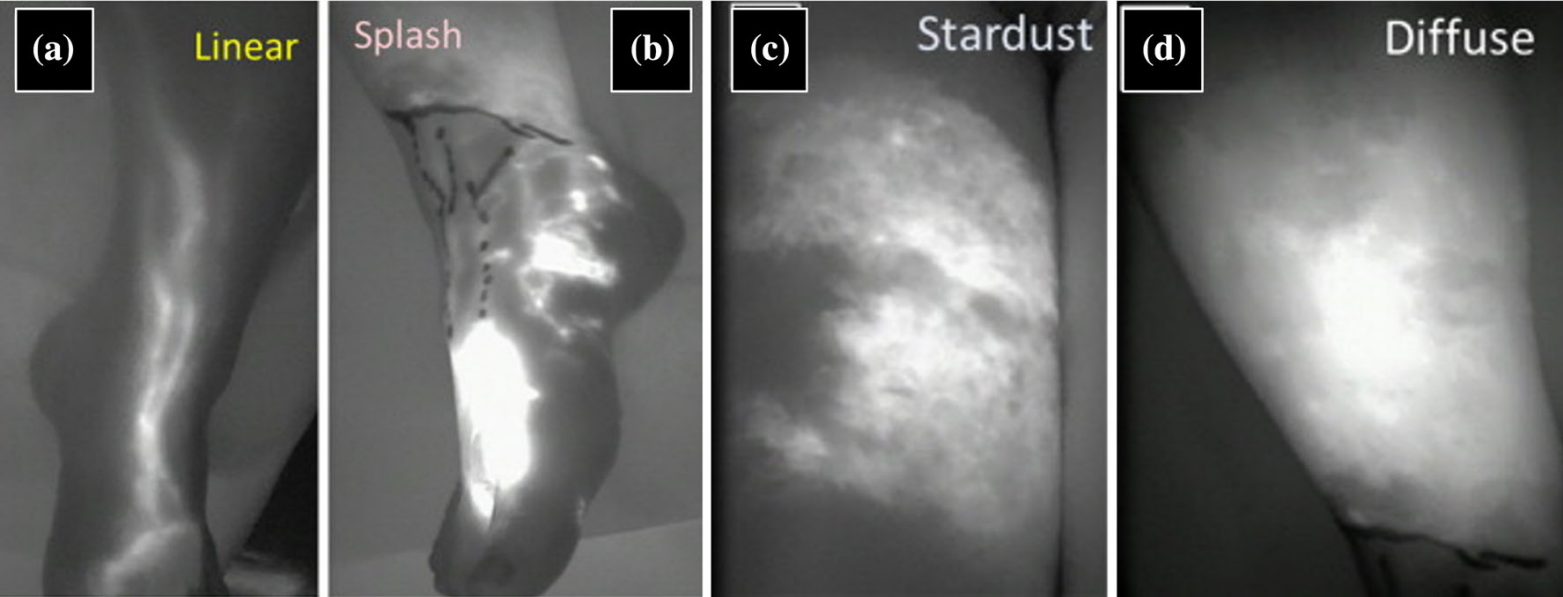
Near-infrared fluorescence lymphography images. The images represent normal and abnormal lymphatic drainage patterns in order of severity (left is normal and right is the most severe dermal backflow pattern). Reprinted from Mihara et al.^95^ with permission from Elsevier

Diagnosis and severity staging were most often based on either the MD Anderson Cancer Center (MDACC) scale^59,65–70^ or the Dermal Backflow Scale (DBS).^61,62,67,71–82^ The MDACC scale focuses on the visualization of patent lymphatic vessels in combination with the presence of DBF. In contrast, the DBS focuses on the proximal to distal extension of different DBF patterns^61,71,75,78,81,82^ (in order of severity: normal, splash, stardust and diffuse) [see Fig. 4]. Both scales have been validated and are reproducible. However, the DBS tends to systematically overestimate severity in the early stages of lymphedema.^67^

The DBS is based on the hypothesis that DBF in secondary limb lymphedema starts proximally and extends distally with lymphedema severity. However, there have been cases where DBF originated distally, suggesting the presence of latent primary hypoplasia, where symptoms were triggered by lymph node dissection.^83^

Both systems look at each limb separately. An approach where laterality (i.e., unilateral or bilateral lymphedema) is taken into account has been proposed for lower limb lymphedema.^84^ Furthermore, a quantitative approach has also been used where the lower extremity is divided into 10 consecutive areas and the most proximal anatomical area the ICG dye reaches after a set amount of time is determined.^85,86^

Multiple studies looked at the relationship between clinical severity and NIRF-L patterns. The DBS had a significant positive correlation with the Campisi scale and lymphedema duration, and indicates which treatment option is appropriate.^71,78^ However, very weak correlations between the MDACC scale and the ISL clinical scale were reported, suggesting that both clinical and NIRF-L assessments are needed for surgical decision making.^65,67^

Circumference differences on multiple sites of the arm, especially the forearm, can also be indicative for abnormal DBF patterns.^70,73,87^ In addition, a lack of increased water content or pitting edema was related to the absence of DBF.^73^ On the other hand, correlations between NIRF-L stages and clinical signs were absent^65^ or weak^67,69^ in other studies.

#### Early Diagnosis

NIRF-L can also be used for regular follow-up after cancer surgery. Detection of early abnormal flow is indicative of subclinical lymphedema and is a key point for early intervention.^75,81^ Advanced DBF patterns have been related to longer lymphedema duration, higher age, and longer time until lymphedema diagnosis, suggesting that early detection is of imminent importance.^67^ Abnormal patterns can even be detected before clinical symptoms are present.^62,76,81^ One study reported increased flow in early-stage patients compared with higher-stage and control subjects, which might be useful for effective drainage after LVB surgery.^85^

#### Quantitative Parameters

In studies investigating quantitative parameters related to lymphatic pump function, similar quantitative parameters to scintigraphy were obtained, such as the TT. Significant correlations between the NIRF-L and scintigraphy values were reported.^88^ There was also a correlation between increase in TT and NIRF-L staging systems.^74,77^ Furthermore, the lymph flow velocity and number of contractions/minute have been obtained using different methods^55,74,77,89–91^ (numerical values are included in electronic supplementary Table 6). Some studies found a significant decrease in flow velocity with the increase in disease severity,^74,77^ while others reported high variability and poor repeatability of the values and no significant correlation between disease severity and flow velocity.^55,91^ This renders clinical decision making based on quantitative parameters difficult. Moreover, both velocity and contractility were influenced by increased temperature and exercise, indicating the need for uniform methodology.^89,91^

#### Surgical Planning

Most studies suggested that NIRF-L is useful for surgical planning but DBF might mask some lymphatic vessels. Predictive lymphatic mapping was proposed as a potential solution in these cases, which is based on the assumption that the lymphatic anatomy is symmetrical between limbs. Relative distances between lymphatic vessels and predefined anatomic landmarks from the healthy limb were mapped to the affected limb to identify potential anastomosis locations, with success.^92,93^

#### Comparison with Lymphoscintigraphy

Significant correlation between the NIRF-L and lymphoscintigraphy staging systems has been reported.^61,66,68,94^ DBF patterns were consistent between the techniques but NIRF-L allowed for more precise demarcation of lymphatic vessels.^59^ The reported range of sensitivity was higher or similar for NIRF-L (89.0–89.5%) compared with lymphoscintigraphy (45–93%), with highly variable specificities between studies (NIRF-L: 80–86%; lymphoscintigraphy: 26.7–100%).^61,95^ NIRF-L is superior to lymphoscintigraphy for early lymphedema diagnosis, with a sensitivity and specificity of 76% and 80% for NIRF-L and 11% and 0% for lymphoscintigraphy, respectively.^94,95^

#### Injection and Imaging Protocols

Generally, ICG was injected in the interdigital spaces (electronic supplementary Table 5); however, some studies investigated the advantages of multi-lymphosome injections.^72,79,80,96,97^ Multiple ICG injections are possible due to the low risk, limited toxicity, and absence of radiation exposure concerns.^54^ The added value of multi-lymphosome injection lies within the preoperative selection for LVB sites, yielding significantly better postoperative results because more functional lymphatic vessels were detected.^79^ Functional vessels were more often seen around linear, splash, and stardust patterns.^72^ Additionally, a multi-lymphosome-based severity classification system was proposed but one injection is sufficient for DBF evaluation.^80,96,97^

### Magnetic Resonance Lymphography (MRL)

MRL provides high-resolution imaging of large body surface areas. It facilitates in choosing the appropriate surgical or conservative treatment used for therapeutic outcome evaluation.^98^ Electronic supplementary Table 7 gives an overview of the MRL study characteristics.

Because of the versatility of MRI, multiple sequences were deployed to assess different lymphedema properties. Heavily T2-weighted images were acquired before contrast agent injection, to assess soft tissue changes and fluid accumulation in the subcutaneous tissue.^98–111^

**Fig. 5:**
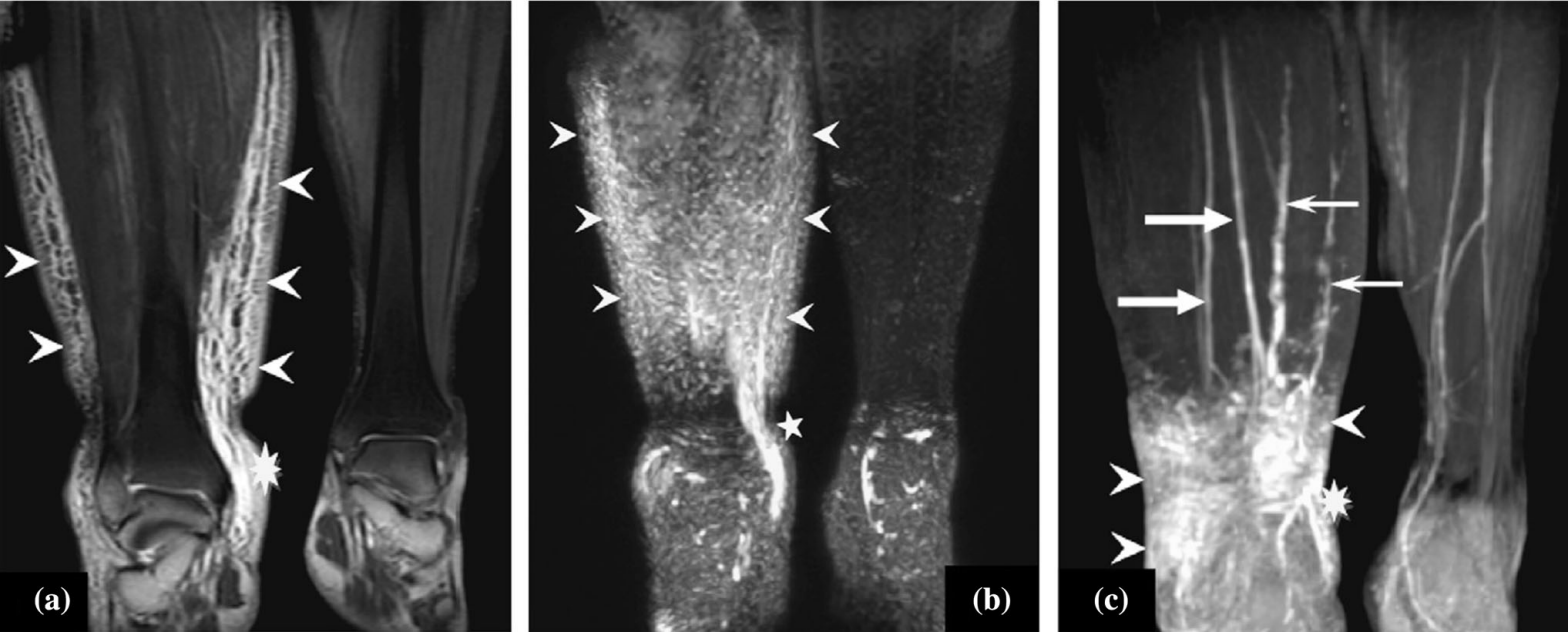
**a** Coronal T2-weighted 2D-TSE image with fat suppression shows an extensive reticular pattern of dilated lymphatic vessels, indicating neovascularization due to obstruction in the right lower leg (arrowheads). **b** Frontal 3D heavy T2-weighted MIP image demonstrates the same changes in the right lower leg (arrowheads). **c** Frontal 3D spoiled gradient-echo T1-weighted MRL MIP image obtained 35 min after Gd-BOPTA injection. Two slightly enlarged lymphatic vessels are visualized in the affected right lower leg (small arrows). The concomitantly enhanced veins (large arrows) show lower signal intensity. Furthermore, areas of accumulated lymph fluid are detected in the three modalities image (asterisk). No lymphedema is seen in the left lower leg. *2D-TSE* two-dimensional turbo spin-echo, *3D* three-dimensional, *MRL* magnetic resonance lymphography. Reprinted from Lu et al.^162^ with permission from Elsevier

Subsequently, T1-weighted sequences with fat suppression were used to visualize the contrast agent uptake in the lymphatic vessels. Maximum intensity projections from any arbitrary plane were obtained for image assessment (see Fig. 5 for MRL images). Electronic supplementary Table 8 shows the imaging protocol information of the MRL studies.

#### Contrast-Enhanced MRL: Parameters for Diagnosis

Contrast-enhanced MRL (CEMRL) uses an intracutaneous injection of a gadolinium-based contrast agent and makes the visualization of superficial and deep lymphatic vessels,^100–102,104,107,111,112^ lymphatic collaterals, DBF, and lymphorrhea possible.^100–102,104,106,107,113,114^ Higher resolution, better fat suppression and signal-to-noise ratio for the lymphatic vessels can be obtained with higher field strengths.^110^

Gadolinium-based contrast agents are not specifically lymphotropic and therefore simultaneously enhance the lymphatic vessels and veins.^108,110^ Studies distinguished these by their morphological or contrast agent uptake and clearance differences. Affected lymphatic vessels had a beaded and tortuous appearance in contrast to smooth veins.^98,100–102,104,106,107,111,112,115^ Moreover, blood had a significantly faster uptake and clearance rate, leading to earlier enhancement and faster decreases of image intensity compared with lymphatic vessels.^102,107,111,115,116^ Similar to lymphoscintigraphy and NIRF-L, severity staging has been proposed on the extent of DBF and visualized lymphatic vessels, which was significantly related to the ISL clinical scale.^112^ Lymphatic vessels in the lymphedematous limb also had an increased diameter compared with healthy vessels but were smaller than the subcutaneous veins. Morphological features of the lymphatic vessels identified with MRL also correlated significantly with immunohistological findings of the corresponding vessels.^115,117^ However, it was not always possible to differentiate lymphatic vessels based on their morphological features^100^ and there was low agreement on judgment of the level of venous contamination between different observers.^103^ Enhancement kinetics was especially important in these cases.^115^ Subcutaneous injection can even lead to solely venous enhancement, rendering lymphedema diagnosis impossible.^99,110^ Dual-agent relaxation MRL uses intravenous administration of ferumoxytol prior to imaging to null the venous signal, and eliminated venous enhancement in the vast majority of cases.^103^ However, the downside of this technique is subsequent signal suppression in the lymphatic channels also, leading to a decreased contrast-to-noise ratio.

The T1-weighted MRL sequences also suffer from T2* susceptibility artifacts in locations of high gadolinium concentrations, such as the injection sites, but are minimal outside the injection sites.^101,102,110,116^ Using fast spin echo instead of gradient-recalled echo sequences can also reduce vulnerability to susceptibility artefacts and field inhomogeneities.^118^

#### Lymphatic Vessel Diameter and Diagnosis

Correlations between MRL findings and clinical severity in secondary lower limb lymphedema have been reported. The number of visualized lymphatic vessels in the calf, and their diameter, was indicative of the clinical severity. This was not the case for the lymphatic vessels in the thigh.^119^ However, lymphatic vessel diameters in the calf and thigh were significantly higher in the affected limb compared with the healthy limb.^115,119,120^ Multiple studies failed to visualize healthy lymphatic vessels because of their small diameter, and reported that only dilated lymph vessels could be clearly depicted on the images.^98,106,115,120,121^ Vessels were also more easily depicted in the lower leg compared with the thigh.^100,102,107^

#### Comparison with NIRF-L

Multiple studies showed the potential of MRL in surgical planning in comparison with NIRF-L. MRL was a reliable tool for identifying potential anastomosis locations, with a sensitivity and specificity of 90% and 100%, respectively. In some cases, the treatment plan was altered (e.g., additional liposuction) due to findings (e.g. fat hypertrophy) not detected with NIRF-L.^109^ More lymphatic vessels were detected with MRL, probably because MRL can also visualize deeper vessels and does not suffer from DBF coverage, making it more sensitive for lymphatic vessel detection.^122–124^ However, only 57.1% of the anastomosis sites located solely with MRL were successful. This percentage was substantially higher when lymphatic vessels were identified with both NIRF-L and MRL, namely 91.4%.^122^ MRL detected LVB sites did result in better postoperative results compared with anastomosis sites selected with NIRF-L.^124^ Lastly, MRL can visualize communicating lymphatic perforators between the deep and superficial lymphatics^125^ and collateral pathways,^114^ which might influence surgical planning.

#### Comparison with Lymphoscintigraphy

Multiple studies investigated the differences between MRL and lymphoscintigraphy. MRL has a better interobserver agreement^126^ and was better at depicting lymphatic vessels due to the substantially better resolution and the ability to look past DBF.^99,108^ In line with these results, very poor correlation was reported for the detection of lymph vessels between these techniques, while excellent correlation was found for observation of drainage delay and drainage patterns.^99,108,126^ MRL seemed less suitable for abnormal lymph node detection.^108^ Lastly, MRL was inferior to scintigraphy as a diagnostic method based on DBF visualization.^105^

#### Non-contrast MRL: Parameters for Diagnosis

Non-contrast magnetic resonance lymphangiography (NCMRL) uses T2-weighted sequences to visualize slow-moving fluid combined with suppression of signal from other tissues. Multiple studies used changes of the dermis and subcutaneous tissue, such as presence of a honeycomb pattern, dermal thickening, and reduction of muscular trophism, for diagnosis and severity assessment.^126–129^ Visualization of the lymphatic vessels was unsuccessful or played a minimal role in NCMRL assessment of lymphedema.^128,130^ In some studies, dilated lymphatic vessels were detected in the affected limb,^129^ and indeed, the presence of dilated vessels was related to clinical severity.^127^ However, lymphatic vessel detection was limited due to the relatively low resolution of NCMRL.^127,129^

#### Positron Emission Tomography/Magnetic Resonance (PET/MR)

Two studies reported on combined positron emission tomography/MR (PET/MR) imaging for lymphedema diagnosis and surgical planning. Subcutaneous injection of ^68^Ga-NOTA-Evans Blue (NEB) allows for visualization of the lymphatic vessels with relatively fast uptake speeds. Both studies reported that combined PET and MR assessment allows for both quantitative (standard uptake value, tracer transport delays) and qualitative assessment of lymphedema severity in three dimensions (DBF, subcutaneous layer thickness)^131,132^ as well as its potential for surgical planning.^131^

### Ultrasound

High frequency ultrasound devices facilitate detailed real-time visualization of lymphatic vessels and veins. Conventional high frequencies (CHFUS) of between 15 and 24 MHz^133–140^ and/or ultra-high frequencies (UHFUS) of between 48 and 70 MHz were used.^141,142^ Electronic supplementary Table 9 gives an overview of the study characteristics.

#### Parameters for Lymphatic Vessel Detection, Diagnosis, and Severity Staging

Lymphatic vessels were detected based on their appearance on the ultrasound image, and were identified after a process of eliminating veins and nerves. Differentiation of lymphatic vessels from other structures was based on shape,^133–135,137,139,141–143^ echogenic texture,^133–135,137,139,141–143^ Doppler color,^133–135,137–139,141–143^ collapsibility,^134,138,139,141,142^ convergence,^138,139,141,142^ and location.^138^ The findings of the first four criteria differed depending on the severity of sclerosis.^134,141^

**Fig. 6:**
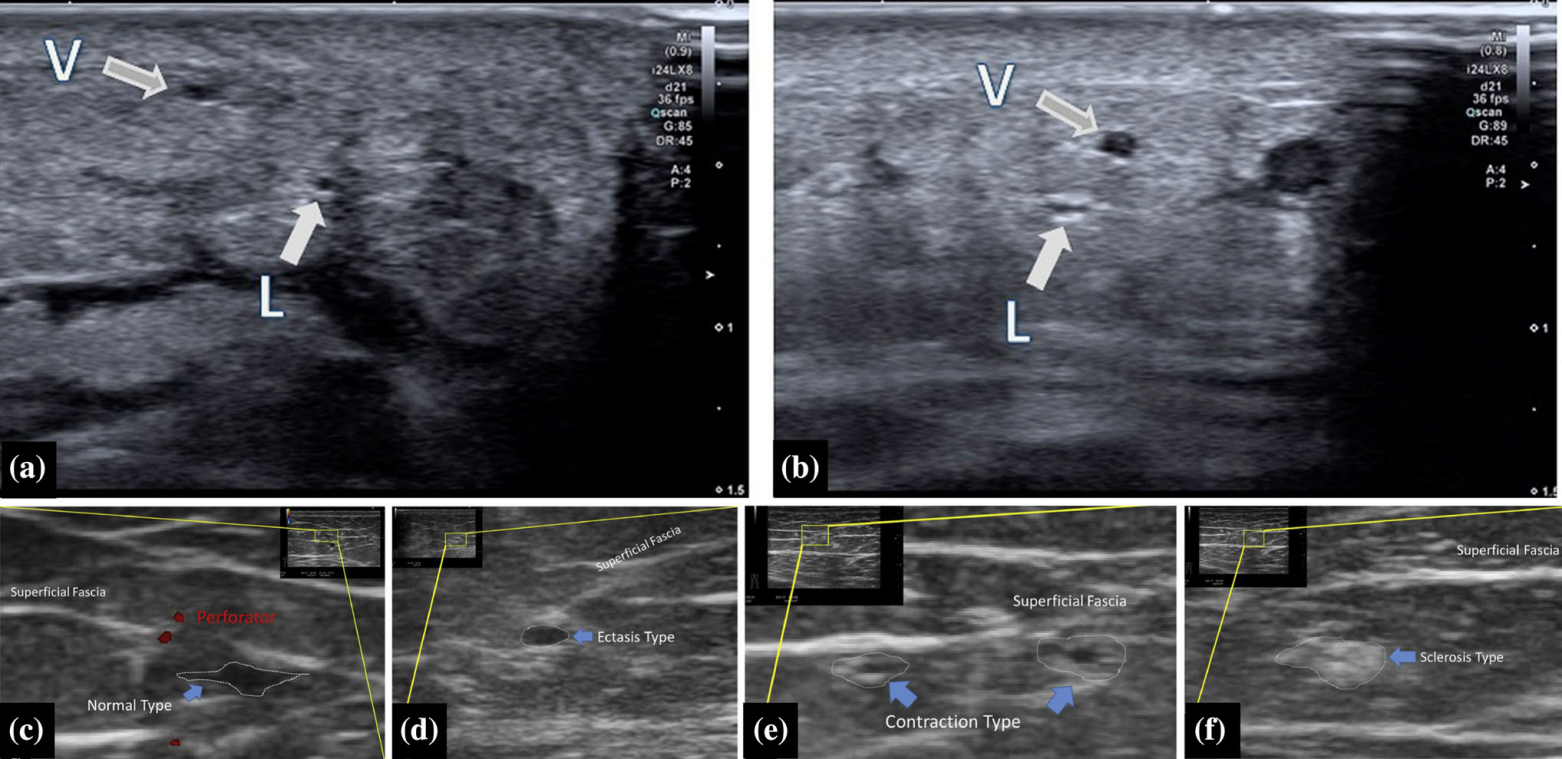
**a, b** Ultrasonographic images of veins (V) and lymphatic vessels (L). Reprinted from Czedik-Eysenberg et al.^137^ with permission from John Wiley & Sons, Inc. **c, d, e, f** Ultrasonographic images of different lymphatic vessel types according to the NECST classification. Reprinted from Mihara et al.^134^ with permission from Elsevier

Lymphatic vessels were also classified into different types based on the degree of degradation; namely, normal, ectasis, contraction, or sclerosis type,^134,140^ or type I (normal + ectasis) and type II (contraction + sclerosis).^142^ The goal of differentiating between these types was optimal vessel selection for LVB surgery (i.e., ectasis-type vessels)^134,140^ or diagnosis.^138^ Vessels with a dilated lumen (ectasis type) or the presence of sclerosis (contraction and sclerosis type) were diagnosed as lymphedema, with a sensitivity, specificity, and accuracy of 95.0%, 100%, and 94.6% respectively.^138^ Figure 6 shows example ultrasound images.

#### Lymphatic Vessel Detection Performance

The majority of the studies reported on vessel detection performance with different gold standards (electronic supplementary Table 10). Overall, sensitivities ranging from 66.3 to 95.5% for lymphatic vessel detection were reported,^133–135,141^ with higher sensitivities for ectasis-(82.9%), contraction- (85.7%), and sclerosis-type (85.7%) vessels in contrast to normal-type (66.7%) vessels.^134^ Overall, detection sensitivity was also higher with UHFUS (94.9%) compared with CHFUS (66.3%).^141^ However, the accuracy of the vessel classification was below 50% for normal, contraction and sclerosis type vessels and was 62.9% for ectasis type vessels. Specificities ranged between 91.3% and 100%, with a higher specificity for UHFUS (98.8%) compared with CHFUS (91.3%).^141^ Sometimes more suitable lymphatic vessels for anastomosis were detected with ultrasound compared with NIRF-L.^137,138,144^

#### Vessel Diameter and Depth

Lymphatic vessel diameters were mostly reported in the leg, ranging from 0.417 to 1.15 mm; lymphatic vessels of the arm were smaller.^141^ Changing body position from supine to sitting or standing also caused a decrease in diameter.^136^ Vessel diameters found with CHFUS were significantly larger than with UHFUS.^141^ Moreover, larger and more vessels were detected with ultrasound compared with NIRF-L.^140^ Post-surgery circumference reduction was significantly higher in this group.^135^ Lastly, vessel measurements significantly correlated between ultrasound and histology measurements.^142^

The maximum depth of lymphatic vessels found depended on the location^135,137^ and frequency used.^141^ Lymphatic vessels that run more deeply in the upper arm and thigh were more difficult to visualize, especially with 70 MHz probes. Frequencies up to 48 MHz are sufficient for visualization of deeper vessels.^142^

#### Photoacoustic Imaging

**Fig. 7:**
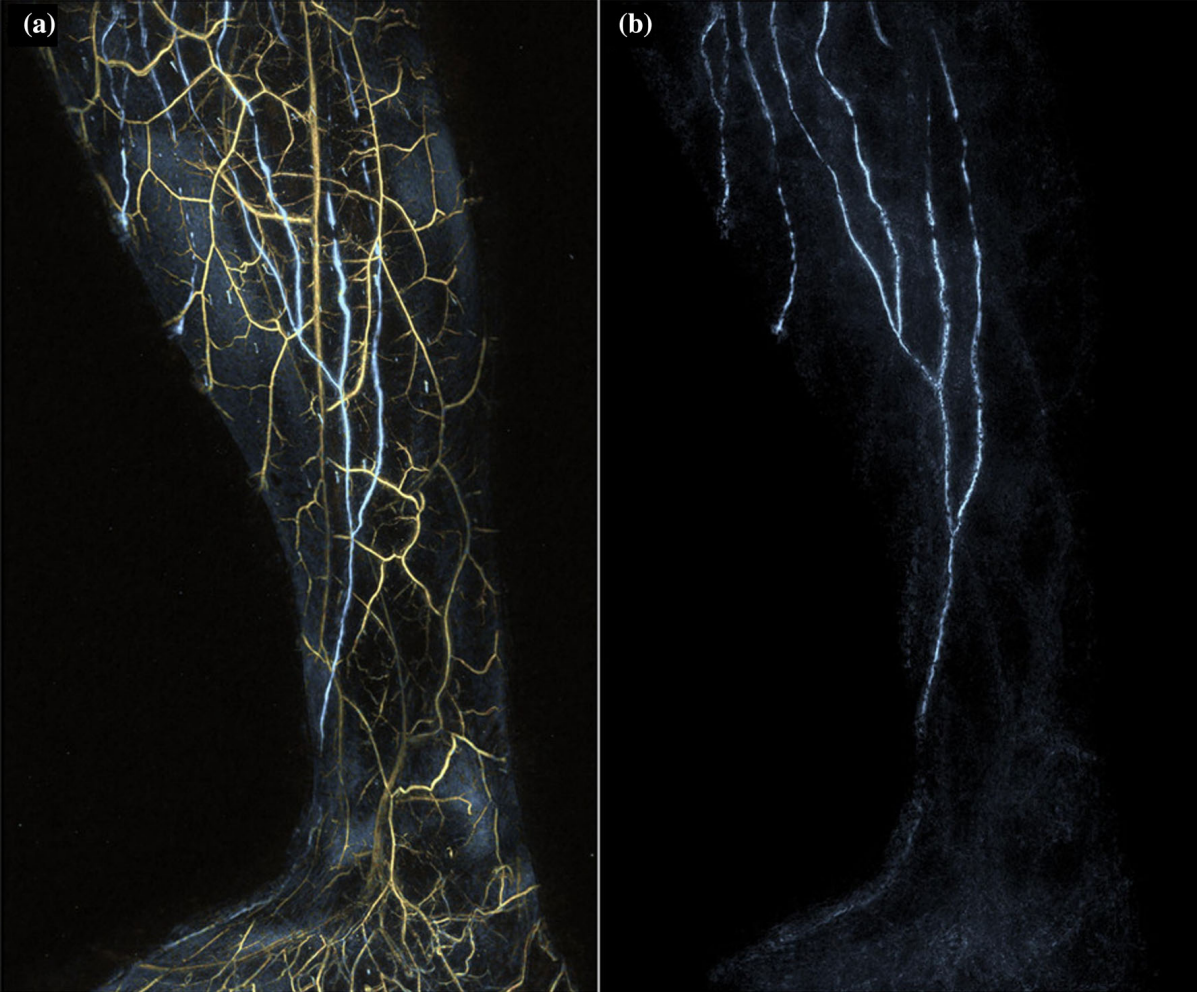
Photoacoustic images. The medial-side view of the photoacoustic lymphangiography of the right lower leg of a woman in her thirties without any past medical history. **a** Lymphatic vessels are shown in blue and venules are shown in yellow. **b** Only lymphatic vessels are shown. Reprinted from Kajita et al.^145^ with permission from John Wiley & Sons, Inc

PAI is a new modality not yet used in clinical practice. It also uses ICG but depends on its optical absorption properties. Light of the specific wavelength is absorbed by chromophores such as melanin, hemoglobin, or ICG, causing thermoelastic expansion and generating acoustic waves detected with an ultrasound transducer. Studies showed that 3D high-resolution imaging and differentiation of lymphatic and blood vessels is possible along with DBF characterization.^145–150^ Figure 7 shows example PAI images.

Lymphedema severity classification was proposed based on visualization of lymphatic collectors, precollectors, and capillaries.^148,150^ PAI findings corresponded well with NIRF-L^148,150^ but more lymphatic vessels were identified using PAI. Furthermore, functioning lymphatic vessels were identified with PAI in locations with diffuse NIRF-L patterns.^150^ PAI also seemed to be less affected by thicker subcutaneous tissue^146^ but was less sensitive for ICG in the interstitium due to dilution.^148,150^ Lastly, the imaged anatomical morphology has also been successfully matched to the surgical field.^149^

## Discussion

(Super)microsurgical treatment planning of lymphedema critically depends on the imaging technique. The ideal imaging modality can detect functional lymphatic vessels, shows their location in three dimensions, and displays the venous network. With this systematic review, we provide an overview of the existing imaging modalities used for preoperative visualization of the lymphatic vessels.

A wide variety of imaging modalities are available for lymphedema diagnosis, severity staging, and surgical planning. NIRF-L is superior to lymphoscintigraphy in lymphatic vessel depiction for surgical planning. Lymphoscintigraphy provides two-dimensional visualization in a large field of view but the wide variety in imaging protocols suggest that there is no consensus on the optimal method.^151^ The main disadvantage is the low resolution, which makes clear depiction of lymphatic vessels, and therefore precisely locating anastomosis sites, unreliable.^25,36–38,41^ Other disadvantages are the lack of in-depth information and the long acquisition duration. SPECT/CT could offer a significant advantage for 3D localization but remains understudied and is not routinely used in practice. Lymphoscintigraphy and SPECT/CT impose radiation exposure, while NIRF-L is not associated with ionizing radiation and ICG has an excellent safety profile.^54^ Moreover, NIRF-L has superior image resolution and provides real-time imaging of lymphatic vessels and vessel contractions for intuitive evaluation.^56,59,82^ Imaging assessment methods are also more uniform and suitable for (early) diagnosis.^62,67,75,76^ The downside of NIRF-L is the absence of in-depth information on lymphatic vessels and the limited depth penetration (approximately 1–2 cm).^152,153^ The appearance of lymphatic vessels changes due to optical scattering and saturation of the camera for superficially pooled ICG, possibly masking deeper targets.^68,78^ Lastly, visualization of the acceptor veins is not possible.

MRL provides 3D high-resolution simultaneous lymphatic vessel and vein enhancement, which has the advantage that LVB sites can be selected, but it can also lead to misidentification and thus inaccurate surgical planning.^100,103^ Moreover, non-dilated vessels are often not visible, limiting early diagnosis based on MRL findings.^98,106,120,121^ MRL is also less practical for routine implementation in secondary lymphedema due to the limited availability and high costs, which leads to logistical challenges to do surgical planning with up-to-date images and makes regular follow-up with MRL unrealistic. When a more detailed overview of the entire lymphatic system is needed, such as in primary lymphedema cases, MRL is indicated.^154–156^

Contrarily, clinical implementation of HFUS is less tedious due to its portability. HFUS is also complemented by the ability to make accurate diameter measurements of both lymphatic vessels and veins.^139^ HFUS provides selection of optimal lymphatic vessels and veins based on their morphological appearance, which may improve LVB surgical outcomes.^157,158^ Additionally, the technique is label-free and is not influenced by DBF.^133,135,142^ The major downside is the high operator dependency and the demanding learning curve. Implementation of this technique is therefore not straightforward.^133,135,137,141^

Finally, the properties of PAI make simultaneous visualization and differentiation of the lymphatic vessels and veins with a high 3D spatial and temporal resolution possible.^159^ This might overcome problems with misidentification of structures and the lack of in-depth information. PAI thus fulfills many of the criteria for an ideal imaging modality for surgical planning. The downside of the photoacoustic devices in the current studies is the large size of the imaging system and the use of high-power lasers. Portable and LED-based systems have been developed, making clinical implementation safer and easier,^160,161^ although the lower optical power limits penetration depth to about 1 cm.

Clinical use of the acquired images pivots on the definition of disease scales, which rely on counting or scoring of image parameters. A common aspect of all modalities included here is that interpretation, annotation, and measurement of the images by a human expert is critical. This manual process is time-consuming and prone to individual variability, undermining the robustness of scoring systems. Another limitation that is shared between all imaging techniques except ultrasound is the use of exogenous contrast. Lastly, portable systems used for NIRF-L, US, and handheld PAI enable imaging in the surgical position, limiting the influence of body position on the location and size of the vessels.

This systematic review has some limitations. Due to the wide scope of this review, a heterogeneous group of studies and study populations was included. Very few articles directly compared imaging modalities quantitatively but mostly describe their findings narratively. However, the emphasis of this review was to highlight the imaging techniques and their applications. Second, the search terms were truncated to only find studies limited to imaging of the extremities or head and neck region. Relevant studies may have been missed if they did not mention one of these terms in their keywords, title, or abstract. Lastly, systematic reviews are subject to publication and selection bias, as studies with negative or undesirable results might not be published. We expect that our systematic approach minimized this bias.

## Conclusion

We reviewed six imaging techniques for mapping secondary lymphedema. A wide variety of modality-specific parameters and staging systems are in use. NIRF-L has gained popularity in recent years, in comparison with lymphoscintigraphy, due to its superior image quality and ease of use. It can be usefully compounded with high frequency ultrasound, which also characterizes vessel condition. MRL has been intensely researched for its 3D imaging capability but exhibits limited sensitivity for small structures and remains expensive. Lastly, PAI is a novel technique that capitalizes on a combination of optical and acoustic contrast, visualizing both lymphatic vessels and veins in 3D. More evidence is needed to evaluate the utility of PAI in surgical planning.

## Supplementary Information

Below is the link to the electronic supplementary material.

Supplementary file1 (DOCX 432 kb)
