# Correction: Imaging of the Lymphatic Vessels for Surgical Planning: A Systematic Review

**DOI:** 10.1245/s10434-022-12660-4

**Published:** 2022-10-16

**Authors:** Saskia van Heumen, Jonas J. M. Riksen, Wichor M. Bramer, Gijs van Soest, Dalibor Vasilic

**Affiliations:** 1 Department of Plastic and Reconstructive Surgery, Erasmus MC, University Medical Center, Rotterdam, The Netherlands; 2 Department of Cardiology, Erasmus MC, University Medical Center, Rotterdam, The Netherlands; 3 MSc Educational Program Technical Medicine, Leiden University Medical Center, Delft University of Technology and Erasmus MC, University Medical Center, Rotterdam, The Netherlands; 4 Medical Library, Erasmus MC, University Medical Center, Rotterdam, The Netherlands

## Correction to: Annals of Surgical Oncology 10.1245/s10434-022-12552-7

In the original online version of this article Saskia van Heumen's given name was misspelled.

The original article was corrected.

