# Supplementary material for: Imaging of the Lymphatic Vessels for Surgical Planning: A Systematic Review
Source: Ann Surg Oncol. 2022 Sep 28;30(1):462–79. doi: 10.1245/s10434-022-12552-7 (PMC9726677; doi:10.1245/s10434-022-12552-7)
Supplement: Supplementary file 1 — Supplementary file1 (DOCX 432 kb) [file 10434_2022_12552_MOESM1_ESM.docx]

# Supplemental files

Supplementary table 1: Search query

| Embase | Records |
| --- | --- |
| ('lymph vessel'/exp OR 'lymph flow'/de OR lymphography/de OR 'lymphatic system'/de OR lymphangiography/exp OR lymphoscintigraphy/exp OR ((lymph* NEAR/3 (vessel* OR venule* OR flow* OR function* OR pump* OR vascul* OR channel* OR contractil* OR transport* OR system*)) OR lymphogra* OR lymphangiogra* OR lymphoscintigra*):Ab,ti) AND (imaging/mj/de OR 'diagnostic imaging'/mj/exp OR 'diagnostic imaging equipment'/mj/exp OR 'nuclear magnetic resonance'/mj/exp OR radiodiagnosis/mj/exp OR spectroscopy/mj/exp OR scintigraphy/mj/exp OR dye/mj/exp OR fluorescence/mj/exp OR radioisotope/mj/exp OR echography/mj/exp OR photoacoustics/mj/exp OR technetium/mj OR 'contrast medium'/mj OR (imaging OR radiodiagnos OR Scintigra* OR lymphoscintigra* OR dye OR icg OR indocyanine-green OR fluorescen* OR radioisotope* OR technetium* OR spect OR tomograph* OR ct OR mri OR (magnet* NEAR/3 resonan*) OR Visualization* OR Visualisation* OR Lymphangiogra* OR Lymphogra* OR Mapping OR Quantitat* OR Quantif* OR detect* OR locating* OR echogra* OR ultraso* OR photoacoustic* OR optoacoustic* OR photo-acoustic* OR opto-acoustic* OR 99-tc OR 99tc OR spectroscop* OR near-infrared* OR near-infra-red* OR contrast-medi*):ti) AND (lymphedema/exp OR 'lymphaticovenular anastomosis'/de OR 'lymphovenous anastomosis'/de OR 'lymph node dissection'/de OR (lymphedem* OR lymph-oedem* OR lymph-edem* OR lymphoedem* OR ((Lymphaticovenul* OR Lymphoven* OR Lympho-ven*) NEAR/3 (shunt* OR bypass* OR anastomo*)) OR (lymph-node NEAR/3 dissect*) OR lymphadenectom*):ab,ti) AND (limb/exp OR 'limb disease'/exp OR head/exp OR neck/exp OR 'head and neck tumor'/exp OR (limb OR extremit* OR arm OR arms OR leg OR legs OR foot OR feet OR hand OR hands OR head OR neck):ab,ti) NOT ([animals]/lim NOT [humans]/lim) NOT [conference abstract]/lim NOT [letter]/lim NOT [note]/lim NOT (review/exp OR 'meta analysis'/de OR (review OR meta-analys*):ti) | 1083 |
| Medline All via Ovid |  |
| (Lymphatic Vessels/ OR Lymphography/ OR Lymphatic System/ OR Lymphoscintigraphy/ OR ((lymph* ADJ3 (vessel* OR venule* OR flow* OR function* OR pump* OR vascul* OR channel* OR contractil* OR transport* OR system*)) OR lymphogra* OR lymphangiogra* OR lymphoscintigra*).ab,ti.) AND (exp * Diagnostic Imaging/ OR exp * Magnetic Resonance Imaging/ OR exp * Radiography/ OR exp * Spectrum Analysis/ OR * Coloring Agents/ OR exp * Fluorescence/ OR * Radionuclide Imaging/ OR Radioisotopes/ OR exp * Ultrasonography/ OR * Technetium/ OR * Contrast Media/ OR (imaging OR radiodiagnos OR Scintigra* OR lymphoscintigra* OR dye OR icg OR indocyanine-green OR fluorescen* OR radioisotope* OR technetium* OR spect OR tomograph* OR ct OR mri OR (magnet* ADJ3 resonan*) OR Visualization* OR Visualisation* OR Lymphangiogra* OR Lymphogra* OR Mapping OR Quantitat* OR Quantif* OR detect* OR locating* OR echogra* OR ultraso* OR photoacoustic* OR optoacoustic* OR photo-acoustic* OR opto-acoustic* OR 99-tc OR 99tc OR spectroscop* OR near-infrared* OR near-infra-red*).ti.) AND (exp Lymphedema/ OR Lymph Node Excision/ OR (lymphedem* OR lymph-oedem* OR lymph-edem* OR lymphoedem* OR ((Lymphaticovenul* OR Lymphoven* OR Lympho-ven*) ADJ3 (shunt* OR bypass* OR anastomo*)) OR (lymph-node ADJ3 dissect*) OR lymphadenectom*).ab,ti.) AND (exp Extremities/ OR exp Head/ OR exp Neck/ OR exp Head and Neck Neoplasms/ OR (limb OR extremit* OR arm OR arms OR leg OR legs OR foot OR feet OR hand OR hands OR head OR neck).ab,ti.) NOT (exp animals/ NOT humans/) NOT (Review/ OR Systematic Review/ OR Meta-Analysis/ OR (review OR meta-analys*).ti.) | 782 |
| Web of Science Core Collection |  |
| TS=((((lymph* NEAR/2 (vessel* OR venule* OR flow* OR function* OR pump* OR vascul* OR channel* OR contractil* OR transport* OR system*)) OR lymphogra* OR lymphangiogra* OR lymphoscintigra*)) AND ((lymphedem* OR lymph-oedem* OR lymph-edem* OR lymphoedem* OR ((Lymphaticovenul* OR Lymphoven* OR Lympho-ven*) NEAR/2 (shunt* OR bypass* OR anastomo*)) OR (lymph-node NEAR/2 dissect*) OR lymphadenectom*)) AND ((limb OR extremit* OR arm OR arms OR leg OR legs OR foot OR feet OR hand OR hands OR head OR neck))) AND TI=((imaging OR radiodiagnos OR Scintigra* OR lymphoscintigra* OR dye OR icg OR indocyanine-green OR fluorescen* OR radioisotope* OR technetium* OR spect OR tomograph* OR ct OR mri OR (magnet* NEAR/2 resonan*) OR Visualization* OR Visualisation* OR Lymphangiogra* OR Lymphogra* OR Mapping OR Quantitat* OR Quantif* OR detect* OR locating* OR echogra* OR ultraso* OR photoacoustic* OR optoacoustic* OR photo-acoustic* OR opto-acoustic* OR 99-tc OR 99tc OR spectroscop* OR near-infrared* OR near-infra-red* OR contrast-medi*) NOT ((animal* OR rat OR rats OR murine OR mouse OR mice) NOT (human* OR patient*))) AND DT=(article) NOT TI=(review OR meta-analys*) | 658 |
| Cochrane CENTRAL Register of Trials |  |
| (((lymph* NEAR/3 (vessel* OR venule* OR flow* OR function* OR pump* OR vascul* OR channel* OR contractil* OR transport* OR system*)) OR lymphogra* OR lymphangiogra* OR lymphoscintigra*):Ab,ti) AND ((imaging OR radiodiagnos OR Scintigra* OR lymphoscintigra* OR dye OR icg OR indocyanine NEXT green OR fluorescen* OR radioisotope* OR technetium* OR spect OR tomograph* OR ct OR mri OR (magnet* NEAR/3 resonan*) OR Visualization* OR Visualisation* OR Lymphangiogra* OR Lymphogra* OR Mapping OR Quantitat* OR Quantif* OR detect* OR locating* OR echogra* OR ultraso* OR photoacoustic* OR optoacoustic* OR photo NEXT acoustic* OR opto NEXT acoustic* OR 99 NEXT tc OR 99tc OR spectroscop* OR "near infrared*" OR "near infra red*" OR contrast NEXT medi*):ti) AND ((lymphedem* OR lymph NEXT oedem* OR lymph NEXT edem* OR lymphoedem* OR ((Lymphaticovenul* OR Lymphoven* OR Lympho NEXT ven*) NEAR/3 (shunt* OR bypass* OR anastomo*)) OR (lymph NEXT node NEAR/3 dissect*) OR lymphadenectom*):ab,ti) AND ((limb OR extremit* OR arm OR arms OR leg OR legs OR foot OR feet OR hand OR hands OR head OR neck):ab,ti) | 13 |

Supplementary table 2: Characteristics of included studies on lymphoscintigraphy

| Study information | | Patient characteristics | | | | LE type (No.) | | | Limbs (No.) | |
| --- | --- | --- | --- | --- | --- | --- | --- | --- | --- | --- |
| Year | Author | M | F | Age*  (years) | ISL stage (%) | P | S | H | UL | LL |
|  |  | (No.) | |  |  |  |  |  |  |  |
| Diagnosis, severity staging and treatment indication | | | | | | | | | | |
| 2021 | Bourgeois^1^ | 5 | 7 | 43.3 (21 - 70) | - | 7 | 5 | - | HN: 12 | |
| 2020 | Pappalardo^2^ | - | 141 | 60.2 ± 12.4 (26 - 82) | - | - | 141 | - | - | 141 |
| 2019 | Campisi^3^ | 65 | 183 | 51.2 (9 -84) | - | 151 | 97 | - | 48 | 200 |
| 2018 | Tartaglione^4^ | 96 | | 52 ± 9 | - | - | 96 | - | 58 | 80 |
| 2018 | Cheng^5^ | 19 | 266 | 56.2 ± 14.3 (2 - 89) | - | 30 | 155 | - | 126 | 159 |
| 2017 | Maclellan^6^ | 134 | | 27 (1 – 88) | I: 54 II: 30 III 16 | 106 | 28 | - | 24 | 157 |
| 2017 | Hassanein^7^ | 64 | 163 | 36.7 ± 23 | - | 116 | 45 | 58 | 227 | |
| 2015 | Yoo^8^ | - | 72 | 53.0 ± 8.1 | - | - | 72 | - | 72 | - |
| 2014 | Devoogdt^9^ | - | 10 | 50 (30 – 67) | - | - | 10 | - | 20 | - |
| 2012 | Kalawat^10^ | 10 | 14 | 47 (13 - 74) | I: 15 II: 5  III: 30 IV: 50 | Unclear etiology | | | - | 48 |
| 2012 | Infante^11^ | 16 | 44 | 43 (1 – 84) | - | 32 | | 28 | 4 | 56 |
| 2011 | Mikami^12^ | 1 | 77 | 55.5 ± 13.2 (22 - 84) | I: 43.6 II: 53.8  III: 2.6 | - | 78 | - | 78 | - |
| 2010 | Maegawa^13^ | 9 | 102 | 63 (38 - 92) | I: 15.8 II: 69.8  III: 14.4 | - | 111 | - | - | 142 |
| 2008 | Pecking^14^ | 4328 | | (23 – 78) | 0: 2.6 I: 12  II: 58 III: 27.4 | NR | | - | - | 4328 |
| 2008 | Dabrowski^15^ | 67 | | 58 (11 – 87) | - | 67 | | 37 | - | 104 |
| 2000 | Williams^16^ | 700 | | - | - | NR | | - | 700 | |
| Predictive value | | | | | | | | | | |
| 2021 | Kwon^17^ | 1 | 16 | 42.1 ± 11.0 | II: 58.8 III: 41.2 | 5 | 12 | - | - | 17 |
| 2021 | Kim^18^ | 9 | 124 | 55.3 (18 – 84) | - | 3 | 130 | - | 64 | 69 |
| 2019 | Kim^19^ | - | 80 | 51.2 ± 10.3 (30 - 75) | I: 36.3 II: 50.0  III: 13.7 | - | 80 | - | 80 | - |
| 2017 | Chiewvit^20^ | 3 | 77 | 59.5 (12 - 72) | - | NR | | - | 80 | |
| Tracer injection methodology | | | | | | | | | | |
| 2021 | Bourgeois^21^ | 76 | 23 | 51.4 (13 – 88) | - | 44 | 55 | - | - | 99 |
| 2010 | Tartaglione^22^ | 12 | 32 | - | - | 21 | 23 | - | 15 | 29 |
| 2006 | O'Mahony^23^ | - | 6 | (41 – 67) | - | - | 6 | - | 6 | - |
| 2004 | O'Mahony^24^ | 10 | 2 | (24 – 40) | - | - | - | 24 | 24 | - |
| 2003 | Stanton^25^ | - | 13 | 54.7 ± 6.4 | - | - | 13 | - | 13 | - |
| SPECT-CT | | | | | | | | | | |
| 2021 | Fujiyoshi^26^ | 5 | 107 | 55.7 ± 12.4  (25 – 82) | - | - | 112 | - | - | 143 |
| 2015 | Baulieu^27^ | 39 | 61 | 52 (1 – 86) | - | 100 | | - | 3 | 94 |
| 2013 | Baulieu^28^ | 7 | 34 | 67 (9 – 84) | - | 17 | 5 | - | - | 41 |
| 2007 | Pecking^29^ | 115 | | (23 – 77) | 0: 25.2 I: 74.8 | NR | | - | - | 115 |

**Values are mean (range) or ± standard deviation; M: male; F: female; ISL: International Society of Lymphology; P: primary; S: secondary; H: healthy; UL: upper limbs; LL: lower limbs; LE: lymphedema; NR: not reported; HN: head and neck*

Supplementary table 3: Contrast agents and imaging methods used for lymphoscintigraphy

|  | Tracer administration | | | | | | Acquisition moments (minutes post-injection) | |
| --- | --- | --- | --- | --- | --- | --- | --- | --- |
| Authors | Contrast agent  (^99m^Tc-labeled) | A  (MBq) | Dose  (mL) | Type | Injection site | Rest/stress/massage | Static | Dynamic |
| Bourgeois^1^ | HSA nanocolloid | 81 | 0.2 | sc | Forehead | Massage | - | - |
| Pappalardo^30^ | phytate | 37 | 0.5 | sc | 1^st^ ids feet | Rest | 5 and 120 | - |
| Campisi^3^ | HSA nanocolloid | 30-50 | - | sc & sf | sc: 2^nd^ ids hands and feet  sf: palmar surface hands and feet | Stress | 30 and 60 | - |
| Tartaglione^4^ | nanocolloid | 50-80 | 0.3 | id | UL: 1^st^ + 4^th^ ims  LL: 1^st^ ims + peroneal malleolus area | Massage & stress | 0 (pre-stress), multiple (post-stress), 60 | - |
| Cheng^5^ | pytate | 37 | 0.5 | sc | 2^nd^ ids hands or 1^st^ ids feet | Stress | 5, 120 | - |
| Maclellan^6^ | filtered sulfur colloid | - | - | id | NR | NR | 45, 120, 240 | - |
| Hassanein^7^ | filtered sulfur colloid | 37 | - | id | 2^nd^ and 4^th^ ids hands and feet | NR | 45, 120, 240 | - |
| Yoo^8^ | pytate | 185 | - | sc | 2^nd^ ids hands | Stress | 30, 60 and 120 |  |
| Devoogdt^9^ | HSA nanocolloid | 85 | 0.2 | sc | 1^st^ and 2^nd^ ids hands | Rest & stress | 1, 45 and 116 (injection sites),  120 (axilla), 124 (whole body) | 3.5 (duration of 40 min 10s/frame) |
| Kalawat^10^ | sulfur colloid | 20 | 0.2 | sc | 1^st^ and 2^nd^ ids feet | Rest (stress in some cases) | 0, 60, 240 and 1140 | - |
| Infante^11^ | nanocolloid | 37 | 0.1 | id | 1^st^ and 2^nd^ ids hands or feet | Massage | 30 and 120 | - |
| Mikami^12^ | HSA nanocolloid | 40 | 0.2 | sc | 2^nd^ and 4^th^ ids hands | Rest | 30 and 120 | - |
| Maegawa^13^ | HSA nanocolloid | 40 | 0.2 | sc | 1^st^ and 3^rd^ ids feet | Rest | 30 and 120 | - |
| Pecking^14^ | HSA nanocolloid | - | 0.2 | sc | 1^st^ ids feet | Stress | 60 | 0 (injection sites 30 s/frame) |
| Dabrowski^15^ | HSA nanocolloid | 37 | - | sc | 1^st^, 2^nd^, 3^rd^ and 4^th^ ids feet | Stress | 120 | 0 (injection site)  20 (lymph nodes) |
| Williams^16^ | HSA nanocolloid | 18.5 | - | id | 2^nd^ ids foot or hand | Rest, Massage & stress | 30, 35 and 180/240 | 0 (duration = 15 min) |
| Kwon^17^ | Tin colloid or phytate | 148 |  |  | 1^st^ and 2^nd^ ids feet | Stress | 0, 60 and 120 | - |
| Kim^18^ | phytate | 37 | 0.4 | sc | 2^nd^ and 3^rd^ ids feet | Stress | 30, 60 | - |
| Kim^19^ | phytate | 148 | - | sc | 2^nd^ and 3^rd^ ids hands | Stress | 60 and 120 | *-* |
| Chiewvit^20^ | dextran | 37 | - | sc | 1^st^ and 2^nd^ ids hands or feet | Massage and stress | 15, 30 ,45, 60 and 240 | *­-* |
| Bourgeois^21^ | HSA nanocolloid | 74-111 | 0.2 | sc | 1^st^ ids feet (if no tracer uptake in lymph nodes additional injection in thigh) | Rest, stress & massage | - | 0 (injection sites)  30 (lymph nodes) |
| Tartaglione^22^ | HSA nanocolloid | 50 | 0.3-0.4 | id | 1^st^ ids hands and feet | Massage & stress | 0 (pre-exercise) and until tracer reached lymph nodes (post-exercise) | - |
| O'Mahony^23^ | HIG | 40 | 0.1 | sc/id | 2^nd^ ids hands | - | 10-16, 37-68, 82-95, 108-133, 148-171 | *-* |
| O'Mahony^24^ | nanocolloid OR HIG | 40 | 0.1 | sc/id | 2^nd^ ids hands | - | Sequential images over 120/180 | *-* |
| Stanton^25^ | polyclonal HIG | 35 | 0.2 | im | Forearm | Massage | Every 30-60 min for 5 hours | 0 (duration = 30 min) |
| Fujiyoshi^26^ | HSA | 40 | - | sc | 1^st^ and 4^th^ ids feet | NR | 120 | - |
| Baulieu^27^ | HSA nanocolloid | 111 | - | sc | 1^st^ ids foot or hand | Rest and stress | 40, 240 | 0 (duration = 40 min) |
| Baulieu^28^ | HSA nanocolloid | 111 | 0.2 | sc | 1^st^ ids foot | Rest and stress | 40, 240 | 0 (duration = 40 min) |
| Pecking^29^ | nanocolloid | - | 0.2 | sc | 1^st^ ids feet | Stress | 40 | - |

*HAS: human serum albumin; HIG: human immunoglobulin; UL: upper limb; LL: lower limb; id: intradermal; sc: subcutaneous; sf = subfascial; im: intramuscular; ids: interdigital space; ims: intermetacarpal space; A: activity*

Supplementary table 4: Characteristics of included studies on near-infrared fluorescence lymphography

| Study information | | Patient characteristics | | | | LE type (No.) | | | Limbs (No.) | |
| --- | --- | --- | --- | --- | --- | --- | --- | --- | --- | --- |
| Year | Author | M | F | Age*  (years) | ISL/*Campisi* stage (%) | P | S | H | UL | LL |
|  |  | (No.) | |  |  |  |  |  |  |  |
| Diagnosis, severity staging and surgical planning | | | | | | | | | | |
| 2022 | Tokumoto^31^ | - | 155 | 57.7 ± 12.2 (28 – 85) | I: 7.8 II: 54.8  III: 34.8. IV: 2.6 | - | 155 | - | 155 | - |
| 2022 | Thomis^32^ | 1 | 127 | 56.7 ± 12.25 (29 – 82) | - | - | 128 | - | 128 | - |
| 2021 | Thomis^33^ | - | 20 | 60.5 ± 7.56 | I: 25 IIa: 55  IIb: 20 | - | 20 | - | 20 | - |
| 2021a | Jørgensen^34^ | - | 200 | 59.46 ± 10.05 | 0: 7.5 I: 18 IIa: 48  IIb: 25.5 III: 1 | - | 200 | - | 200 | - |
| 2021 | Akita^35^ | - | 57 | 58.7 ± 10.4 | Nor: 8.8 0: 25.4  I: 19.3 IIa: 27.2  IIb: 19.3 | - | 57 | - | 26 | 88 |
| 2021b | Jørgensen^36^ | - | 237 | 59.68 ± 9.94 | - | - | 237 | - | 237 | - |
| 2020 | Thomis^37^ | - | 45 | 61.3 ± 9.9 (37 – 82) | I: 20 IIa: 40 IIb: 40 | - | 45 | - | 45 | - |
| 2020 | Medina^38^ | - | 19 | 59 (53 - 68) | IIa: 42.1 IIb: 57.9 | - | 19 | - | 19 | - |
| 2020 | Kinugawa^39^ | 1 | 13 | 70.2 (46 – 86) | - | - | 14 | - | - | 14 |
| 2018 | Lee^40^ | 12 | 3 | 28.3 ± 4.5 (24 – 41) | - | - | - | 15 | - | 15 |
| 2019 | Suami^41^ | - | 100 | 57.73 ± 9.78 | - | - | 100 | - | 103 | - |
| 2019 | Matsumoto^42^ | 4 | 59 | 56.0 (20 – 78) | 0: 23 I: 27  IIa: 28 IIb: 11 | 6 | 55 | - | - | 112 |
| 2019 | Garza^43^ | 22 | 190 | 53.12 ± 11.7 | - | 32 | 180 | - | 130 | 82 |
| 2017 | Shinaoka^44^ | - | 54  7 | 54.8 ± 9.7  58.4 ± 14.1 | 0: 39 I: 37.3  II: 23.1 III: 4.6 | - | 54  - | -  7 | - | 108  14 |
| 2017 | Gentileschi^45^ | 1 | 15 | 58.8 ± 13.1 (27 – 77) | IIa: 43.8 IIb: 56.2 | - | 16 | - | 16 | - |
| 2016 | Tashiro^46^ | 2 | 88 | 59 | 0: 15.5 I: 16.1  II: 51.7 III: 16.7 | - | 90 | - | - | 180 |
| 2016 | Akita^47^ | - | 190 | 56.7 ± 12.4 | - | - | 190 | - | 190 | - |
| 2014b | Mihara^48^ | - | 5 | 53.8 (32 – 72) | IIa: 60 IIb: 40 | - | 5 | - | - | 5 |
| 2014a | Mihara^49^ | - | 72 | 54.5 (25 – 88) | *0: 6.9 I: 15.3*  *II: 26.4. III: 33.3*  *IV: 18.1* | - | 72 | - | - | 144 |
| 2013 | Akita^50^ | - | 100 | 54.8 (32 – 76) | - | - | 100 | - | - | 100 |
| 2012 | Suami^51^ | - | 3  34 | -  54.5 ± 8.5 | - | - | -  34 | 3  - | 3  34 | - |
| 2012 | Aldrich^52^ | 1  1 | 17  5 | (45 – 68)  (26 – 68) | - | - | 18  - | -  6 | 18  6 | - |
| 2011a | Yamamoto^53^ | 1 | 27 | 47.2 (19 - 71) | - | - | 28 | - | - | 56 |
| 2011c | Yamamoto^54^ | - | 20 | 57.5 (35 – 80) | *I: 20 II: 30*  *III: 30 IV: 20* | - | 20 | - | 20 | - |
| 2011b | Yamamoto^55^ | - | 45 | 48 (17 – 73) | *I: 12.8 II: 34.6*  *III: 34.6 IV: 15.4*  *V: 2.6* | 3 | 42 | - | - | 90 |
| 2007 | Unno^56^ | 1  7 | 11  3 | 64.3 ± 13.6  44.9 ± 17.3 | - | - | 12  - | -  10 | - | 12  10 |
| 2020 | Johnson^57^ | - | 23 | 51.6 | - | - | 23 | - | 23 | - |
| 2017 | Tashiro^58^ | - | 29 | 60.5 (36 - 84) | I: 13 II: 80 III: 7 | - | 29 | - | 30 | - |
| Quantitative | | | | | | | | | | |
| 2020 | Kelly^59^ | 10 | - | 25.7 ± 1.3 | - | - | - | 10 | 20 | - |
| 2019 | Granoff^60^ | - | 17 | 60.5 ± 14.2 | - | - | 17 | - | 17 | - |
| 2017 | Groenlund^61^ | 10 | - | (20 – 30) | - | - | - | 10 | - | 20 |
| 2014 | Yamamoto^62^ | - | 15 | 57.5 (41 - 74) | 0: 13.3 I: 20  II: 53.3 III: 13.3 | - | 30 | - | 30 | - |
| 2013 | Yamamoto^63^ | -  - | 12  3 | 23.89 (20.4 - 30.4) | 0: 29.2 I: 29.2  II: 33.3 III: 8.3 | -  - | 12  - | -  3 | - | 24  6 |
| 2010 | Rasmussen^64^ | 1  5 | 19  19 | 49.7 ± 16.7  38.2 ± 11.0 | - | 3  - | 17  - | -  24 | 10  12 | 10  12 |
| 2008 | Unno^65^ | 10  17 | -  - | 33.1 ± 7.9  74.7 ± 7.5 | - | -  Other | | 10  - | - | 10  17 |
| Injection methodology | | | | | | | | | | |
| 2022 | Shinaoka^66^ | 4 | 98 | NR | 0: 28 I: 21.3  IIa: 35.4 IIb: 11  III: 4.3 | - | 84 | 21 | - | 206 |
| 2021 | Hara^67^ | 3 | 102 | 58.9 (20 – 91) | I: 27.6 IIa: 25.2  IIb: 29.1 III: 18.1 | 10 | 95 | - | - | 210 |
| 2020 | Hara^68^ | 7 | 96 | 57.8 (11 - 82) | I: 28.2 IIa: 10.2 IIb: 41.7 III: 5.3 | 10 | 93 | - | - | 206 |
| 2019 | Hara^69^ | 4 | 105 | 60.8 (30 - 88) | I: 32.1 IIa: 24.8  IIb: 35.8 III: 7.3 | 7 | 102 | - | 10 | 186 |
| 2013 | Hara^70^ | 2 | 23 | 54 (25 – 75) | 0: 25.6 I: 7.0  II: 65.1 III: 2.3 | 4 | 21 | - | 2 | 41 |
| Comparison with scintigraphy | | | | | | | | | | |
| 2021 | Yoon^71^ | 14 | 30 | 52.92 ± 15.13 | - | - | 44 | - | - | 44 |
| 2020 | Yoon^72^ | - | 47 | 55.85 ± 10.51 | - | - | 47 | - | 47 | - |
| 2013 | Mihara^73^ | - | 29 | 58.6 (32 – 79) | 0: 22. I: 36.2  IIa: 15.5 IIb: 22.4 III: 3.4 | - | 29 | - | - | 58 |
| 2013 | Akita^74^ | 19 | 115 | 58.5 (9 – 82) | - | 39 | 95 | - | - | 234 |

**Values are mean (range) or ± standard deviation; M: male; F: female; ISL: International Society of Lymphology; P: primary; S: secondary; H: healthy; UL: upper limbs; LL: lower limbs; LE: lymphedema*

Supplementary table 5: Contrast agent administration methods for near-infrared fluoresence lymphography

| Authors | System | Dose (mL) | Type | Contrast medium (ICG) | Injection site | |
| --- | --- | --- | --- | --- | --- | --- |
|  |  |  |  |  | Interdigital space | Other |
| Tokumoto^31^ | PDE | 0.3 | sc | NR | 1^st^ (hand) | - |
| Thomis^32^ | PDE | 0.20 | sc | 0.20% | 1^st^, 4^th^ (hand) | - |
| Thomis^33^ | PDE | 0.20 | sc | 0.20% | 1^st^, 4^th^ (hand) | - |
| Jørgensen^34^ | Hypereye | 0.1 | NR | Verdye 0.25%, Diagnostic Green GmbH | - | ulnar border palmaris longus tendon at wrist level |
| Akita^35^ | PDE | 0.2 | sc | Diagniogreen 0.25%, Daiichi Pharm. | 1^st^ (foot) | 5 sites (hands/feet) |
| Jørgensen^36^ | Hypereye | 0.1 | sc & id | Verdye 0.25%, Diagnostic Green GmbH | 1^st^, 3^rd^ (hand) | ulnar border palmaris longus tendon at wrist level |
| Thomis^37^ | PDE | 0.2 | Id | NR | 1^st^, 4^th^ (hand) | - |
| Medina^38^ | PDE | 0.3 | NR | Verdye 0.5%, Diagnostic Green GmbH | 2^nd^, 4^th^ (hand) | - |
| Kinugawa^39^ | PDE | 0.2 | NR | NR | 1^st^, 4^th^(feet) | lateral and medial ankles |
| Lee^40^ | Moment K | 1 | sc | Dongindang Pharm. | 2^nd^ (hands) | - |
| Suami^41^ | PDE | 0.5 – 1 | id | Verdye 0.5%, Diagnostic Green GmbH | 1^st^, 4^th^ ids hand & | ulnar and volar wrist region |
| Matsumoto^42^ | PDE | 0.05 | id | Diagnogreen 0.5%, Daiichi Pharm. | 1^st^, 4^th^ (feet) | Lateral side foot & posterior side ankle |
| Garza^43^ | PDE | 0.02 | id | Akron Inc. | 1^st^, 2^nd^, 3^rd^, 4^th^ (hand/foot) | radial & ulnar wrist/inferior to the medial and lateral malleoli |
| Shinaoka^44^ | PDE | 0.06  0.04 | sc  id | Diagnogreen 0.625%, Daiichi Pharm.  Diagnogreen 0.625%, Daiichi Pharm. | 1^st^, 4^th^ (foot) | lateral side foot and backside ankle |
| Gentileschi^45^ | PDE | 0.1 | id | Verde Indocianina Pulsion | 2^nd^ (hand) | ulnar border of the palmaris longus |
| Tashiro^46^ | PDE | 0.1 | sc | Diagnogreen 0.5%, Daiichi Pharm. | 1^st^ (feet) | lateral border Achilles tendon |
| Akita^47^ | PDE | 0.3 | sc | NR | 1^st^ (hand) | - |
| Mihara^48^ | PDE | 0.2 | sc | Diagnogreen 0.25%, Daiichi Pharm. | 1^st^ (feet) | lateral border Achilles tendon |
| Mihara^49^ | PDE | 0.2 | id | Diagnogreen 0.5%, Daiichi Pharm. | 1^st^ (feet) | - |
| Akita^50^ | PDE | 0.3 | sc | NR | 1^st^ (feet) | - |
| Suami^51^ | PDE | 0.02 | id | NR | 1^st^, 2^nd^, 3^rd^, 4^th^ (hand) | - |
| Aldrich^52^ | Custom | 0.1 | id | 0.025% | - | Medial, lateral, ventral sides wrist & upper lateral forearm |
| Yamamoto^53^ | PDE | 0.2 | sc | Diagnogreen 0.25%, Daiichi Pharm. | 1^st^ (feet) | lateral border Achilles tendon |
| Yamamoto^54^ | PDE | 0.1 | sc | Diagnogreen 0.25%, Daiichi Pharm. | 2^nd^ (hands) | wrist at ulna |
| Yamamoto^55^ | PDE | 0.2 | sc | Diagnogreen 0.25%, Daiichi Pharm. | 1^st^ (feet) | lateral border Achilles tendon |
| Unno^56^ | PDE | 0.2 | sc | Diagnogreen 0.5%, Daiichi Pharm. | Dorsum of the foot | - |
| Johnson^57^ | PDE | 0.1 | id | 0.0625 %, Akorn Inc. | 1^st^, 4^th^ | volar forearm & cephalic vein (US guided) |
| Tashiro^58^ | PDE | 0.2 | id | Diagnogreen 0.5%, Daiichi Pharm. | 2^nd^ (hand) | ulnar border palmaris longus tendon (wrist level) |
| Kelly^59^ | Custom | 0.1 | id | Nomeco 0.30 % | 2^nd^, 4^th^ (hand) | palmar side wrist |
| Granoff^60^ | PDE | 0.1 | id | ICG with albumin 0.0625% | 1^st^, 4^th^ (hand) | volar forearm & lateral upper ipsilateral arm |
| Groenlund^61^ | Custom | 0.1 | id | Nomeco 0.0305 % | 1^st^, 4^th^ ims (feet) | behind medial malleolus |
| Yamamoto^62^ | PDE | 0.1 | sc | Diagnogreen 0.25%, Daiichi Pharm. | 2^nd^ (hands) | - |
| Yamamoto^63^ | PDE | 0.2 | sc | Diagnogreen 0.25%, Daiichi Pharm. | 1^st^ (feet) | - |
| Rasmussen^64^ | Custom | 0.1 | id | 0.025% | 2 locations (hand/feet) | UL: 2 forearm and 2 upper arms  LL: 2 medial ankles, 1 heel, 2 calf and 1 thigh |
| Unno^65^ | PDE | 0.3 | sc | Diagnogreen 0.5%, Daiichi Pharm. | 2^nd^, 4^th^ (hands) | palmar side wrist |
| Shinaoka^66^ | PDE | 0.2 | sc | Diagnogreen 0.25%, Daiichi Pharm. | 1^st^, 4^th^ (foot) | below the medial malleolus  below the lateral malleolus  midpoint of the straight line that connects the head of the fifth metatarsal bone and the lateral malleolus |
| Hara^67^ | PDE | NR | NR | NR | 1^st^ (feet) | proximal side of the external condyle  mid-lateral point of the proximal end of the patella |
| Hara^68^ | PDE | 0.5 | sc | Diagnogreen 0.5% Daiichi Pharm. | 1^st^ (feet) | proximal side of lateral condyle and lateral side of the superior edge of the knee |
| Hara^69^ | PDE | 0.1 | sc | Diagnogreen 0.5%, Daiichi Pharm. | **Control**: 1^st^ (feet), 2^nd^(hands) | **Multi-injection**: no linear pattern with 1 injection 🡪 also medial or lateral ankle or knee |
| Hara^70^ | PDE | 0.2 | id | Diagnogreen 0.5%, Daiichi Pharm. | 1^st^ (feet) or 2^nd^ (hands) | - |
| Yoon^71^ | Moment K | 0.2 | sc | Diagnogreen 0.25%, Daiichi Pharm. | 1^st^ (feet) | lateral border Achilles tendon |
| Yoon^72^ | Moment K | 0.2 | sc | Diagnogreen 0.25%, Daiichi Pharm. | 1^st^, 3^rd^ (hands) | - |
| Mihara^73^ | PDE | 0.2 | id | Diagnogreen 0.5%, Daiichi Pharm. | 1^st^ (feet) | - |
| Akita^74^ | PDE | 0.3 | sc | NR | 1^st^ (feet) | - |

*PDE: photodynamic eye (Hamamatsu Photonics K.K., Hamamatsu, Japan); sc: subcutaneous; id: intradermal; ims: intermetacarpal space; NR: not reported*

Supplementary table 6: Quantitative near-infrared fluorescence lymphography parameters

|  | | Kelly^59^ | Granoff^60^ | Groenlund^61^ | Yamamoto^62^ | Yamamoto^63^ | Rasmussen^64^ |
| --- | --- | --- | --- | --- | --- | --- | --- |
| **Contractions (min^-1^**) | |  |  |  |  |  |  |
| Arm | *control* | 0.9 ± 0.4 |  |  |  |  | 1.3 ± 1.2 |
|  | *unaffected* |  |  |  |  |  | 1.2 ± 1.0 |
|  | *affected* |  | 1.13  (0.67 – 2.5) |  |  |  | 0.3 ± 0.3 |
| Leg | *control* |  |  | 0.60 ± 0.23 |  |  | 0.4 ± 0.3 |
|  | *unaffected* |  |  |  |  |  | 0.3 ± 0.2 |
|  | *affected* |  |  |  |  |  | 0.2 ± 0.2 |
| **Velocity (cm/s)** | |  |  |  |  |  |  |
| Arm | *control* | 1.1 ± 0.3 |  |  | 0.5 ± 0.3 |  | 0.8 ± 0.4 |
|  | *unaffected* |  |  |  |  |  | 0.8 ± 0.4 |
|  | *affected* |  |  |  | (0.01 - 0.2) |  | 0.7 ± 1.0 |
| Leg | *control* |  |  | 1.51 ± 0.42 |  | 0.2 ± 0.06 | 0.9 ± 0.7 |
|  | *unaffected* |  |  |  |  |  | 0.8 ± 0.5 |
|  | *affected* |  |  |  |  | (0.02 - 0.12) | 0.8 ± 0.4 |

Supplementary table 7: Characteristics of included studies on magnetic resonance lymphangiography

| Study information | | Patient characteristics | | | | LE type (No.) | | | Limbs (No.) | |
| --- | --- | --- | --- | --- | --- | --- | --- | --- | --- | --- |
| Year | Author | M | F | Age*  (years) | ISL stage (%) | P | S | H | UL | LL |
|  |  | (No.) | |  |  |  |  |  |  |  |
| Visualization, diagnosis and severity | | | | | | | | | | |
| 2022 | Soga^75^ | 11 | 45 | 50.9 ± 16.9 | 0: 38.4 I: 18.8  II: 37.5 III: 5.4 | 23 | 89 | - | - | 112 |
| 2021 | Wolfs^76^ | 5 | 5 | 24.6 ± 2.1 | - | - | - | 10 | 10 | - |
| 2021 | Soga^77^ | 11 | 45 | 50.9 ± 16.9 | 0: 38.4 I: 18.8  II: 37.5 III: 5.4 | 23 | 33 | - | - | 112 |
| 2020 | Sheng^78^ | - | 50 | 52.5 (42 - 63) | I: 14 II: 56  III: 30 | - | 50 | - | 100 | - |
| 2020 | Abdelfattah^79^ | - | 59 | - | - | - | 59 | - | 59 | - |
| 2018 | Ripley^80^ | 5  6 | 37  36 | 50.8 ± 14.2  55.4 ± 13.6 | - | - | 42  42 | - | 27  27 | 15  15 |
| 2017 | Gennaro^81^ | - | 20 | 57.6 | - | 3 | 17 | - | 15 | 5 |
| 2016 | Jeon^82^ | 9 | 18 | 39.6 ± 20.5 | - | 5 | 22 | - | 9 | 24 |
| 2016 | Liu^83^ | 335 | 375 | 35 (5 - 78) | II: 89.6 III:10.4 | 378 | 332 | - | NR | NR |
| 2015 | Borri^84^ | - | 3 | (49 – 61) | - | - | 3 | - | 6 | - |
| 2014 | Zhou^85^ | 24 | 37 | 34 ± 17  (5 – 80) | I: 21.9 II: 57.8  III: 20.3 | NR | | | - | 48 |
| 2013 | Liu^86^ | 20 | 25 | 49.2 (11 - 78) | I: 20.9 II: 45.8  III: 33.3 | - | 45 | - | 9 | 36 |
| 2012 | Lu^87^ | - | 40 | 52.3 ± 11.9  (27 - 74) | - | - | 40 | - | - | 48 |
| 2009 | Notohamiprodjo^88^ | 4 | 12 | 44 ± 29 | - | NR | | | - | 16 |
| 2008 | Dimakakos^89^ | 8 | 6 | 38.7 (10 – 66) | I: 66.7 II: 33.3 | NR | | 2 | - | 14 |
| 2007b | Lohrmann^90^ | 3 | 7 | 44 (21 - 64) | - | 8 | 2 | - | - | 20 |
| 2007a | Lohrmann^91^ | 1 | 2 | 53 (22 - 76) | - | 2 | 1 | - | - | 3 |
| 2006c | Lohrmann^92^ | 4 | 8 | 47 (25 - 71) | - | 11 | 1 | - | - | 24 |
| 2006b | Lohrmann^93^ | 2 | 8 | 42 (20 - 79) | - | 8 | 2 | - | - | 10 |
| 2006a | Lohrmann^94^ | 2 | 1 | 60 (54 - 69) | - | 1 | 2 | - | - | 3 |
| Pre-operative LVB planning | | | | | | | | | | |
| 2022 | Yasunaga^95^ | - | 23 | 62.8 ±12.3  (41 – 82) | IIa: 74 IIb: 26 | - | 23 | - | - | 23 |
| 2020 | Yasunga^96^ | 2 | 16 | 57.7 ± 14.2  (30 - 77) | IIa: 70 IIb: 30 | 3 | 15 | - | - | 20 |
| 2019 | Pons^97^ | 5 | 77 | 45.5 | I: 9.8 II: 73.2  III: 17.1 | 21 | 61 | - | 50 | 32 |
| 2018 | Zeltzer^98^ | 1 | 21 | 39.7 (22 - 63) | - | NR | | - | 25 | - |
| 2017 | Mazzei^99^ | 6 | 24 | 30 (18 - 70) | I: 13.3 II: 86.7 | 6 | 24 |  | 13 | 17 |
| Comparison with scintigraphy | | | | | | | | | | |
| 2018 | Bae^100^ | 0  1 | 6  6 | 61 ± 9  28 ± 5 | II: 100  - | -  - | 6  - | -  7 | 6  7 | -  - |
| 2014 | Weiss^101^ | 13 | 42 | 41 (9 – 79) | - | 27 | 18 | - | - | 83 |
| 2012 | Notohamiprodjo^102^ | 9 | 21 | 44 ± 19 | I: 23 II: 62.5  III: 12.5 | NR | | | - | 32 |
| Non-contrast MRL | | | | | | | | | | |
| 2021 | Kim^103^ | 2 | 43 | 58.4 ± 10.5 | 0: 11.1 II: 73.3  Unkown: 28.9 | - | 45 | - | 45 | - |
| 2020b | Cellina^104^ | 25 | 25 | 62 (35 - 77) | I: 44 II: 24  III: 32 | - | 50 | - | - | 75 |
| 2020a | Cellina^105^ | 10 | - | 66.1 ± 5.2  (56 - 75) | - | - | 10 | - | - | 17 |
| 2017 | Cresenzi^106^ | -  - | 25  11 | 62 (44 - 80)  50 (33 – 68) | 0: 56 I-II: 44 | - | 25  - | -  11 | 25 | 36 |
| 2005 | Liu^107^ | 27 | 12 | (3 - 71) | - | 36 | 2 | - | 1 | 37 |
| PET-MR | | | | | | | | | | |
| 2019 | Hou^108^ | - | 13 | 48.6 ± 9.5 | - | NR | | - | - | 13 |
| 2017 | Long^109^ | 2 | 9 | 49.8 ± 12.4 | I: 36 II: 36  III: 28 | NR | | - | - | 22 |

**Values are mean (range) or ± standard deviation; M: male; F: female; ISL: International Society of Lymphology; P: primary; S: secondary; H: healthy; UL: upper limbs; LL: lower limbs; LE: lymphedema; NR: not reported*

Supplementary table 8: Contrast agent administration and imaging methods for magnetic resonance lymphography

| Author | Field (T) | Dose (mL) | Contrast agent | Type | Site | MRL | Acquisition (min post-injection) |
| --- | --- | --- | --- | --- | --- | --- | --- |
| Soga^75^ | 1.5 | 0.5 | Gadoteridol (Prohance) | sc/ic | 1^st^–4^th^ ids feet | T1w 3D DIXON | 5, 16 |
| Wolfs^76^ | 3.0 | 0.5 – 0.7 | Gd-DO3A (Bayer) | ic | 1^st^–4^th^ ids hands | T1w 3D GRE with FS | Before & 1.5, 8.7 and 11.9 |
| Soga^77^ | 1.5 | 0.9 | Gadoteridol (Prohance) | ic | 1^st^–4^th^ ids feet | 3D two-point DIXON | NR |
| Sheng^78^ | 3.0 | 0.9 | Gd-DTPA (Magnevist) | ic | 2^nd^–4^th^ ids hands | T1w 3D GRE with FS | Before, 20 |
| Abdelfattah^79^ | 3.0 | 0.8 | Gd-BOPTA (Multihance) | ic | 1^st^–4^th^ ids hands | T1w 3D GRE with FS | NR |
| Ripley^80^  Gennaro^81^ | 3.0 | 1.0 | Gd-BOPTA (Multihance) | ic | 1^st^–4^th^ ids hands or feet | 3D two-point DIXON | 10, 20, 30, 40, 50, 60 and 70 |
|  |  | 5 mg/kg | Ferumoxytol | iv | intravenous |  |  |
| Jeon^82^ | 1.5 | 0.7-0.8 | Gd-BOPTA (Multihance) | ic | 1^st^–4^th^ ids feet | T1w 3D GRE with FS | Before & 5, 10, 15, 20, 25, 30, 35 and 40 |
| Liu^83^ | 3.0 | < 2 | Gadobutrol (Gadovist) | ic | 1^st^–3^rd^ ids hand(s) or feet | T1w 3D FSE with FS  intermediate-w 3D FSE (VISTA) | NR |
| Borri^84^ | 3.0 | 0.7-0.8 | Gd-BOPTA (Multihance) | ic | 1^st^ and 2^nd^ ids hands/feet | T1w 3D GRE with FS | Before & consecutive time points after |
| Zhou^85^ | 1.5 | 0.9 & 0.02 | Gadoteridol (Prohance) | ic | 1^st^–4^th^ ids hands | T1w 3D GRE with FS | Before & several time post over a period of 45 min |
| Liu^86^ | 3.0 | 0.7-0.8 | Gd-BOPTA (Multihance) | ic | 1^st^–4^th^ ids feet | T1w 3D GRE with FS | Consecutive time points. 6 acquisitions leg (duration = 3 min) and 5 acquisitions in the inguinal region and thigh |
| Lu^87^ | 3.0 | 0.9 | Gd-DPTA (Mangevist) | ic | 1^st^–3^rd^ ids feet | T1w 3D THRIVE with FS | Before and 20 |
| Notohamiprodjo^88^ | 3.0 | 0.9 | Gd-DTPA (Magnevist) | ic | 1^st^–3^rd^ ids feet | T1w 3D FLASH with FS | NR |
| Dimakakos^89^ | 1.5 | 0.9 | Gadobutrol (Gadovist) | sc | 1^st^–4^th^ ids feet | T1w 3D GRE | 5, 10, 15, 30, 45, 60, 90 and 120 |
| Lohrmann^90^ | 1.5 | 1.8 | Gadoteridol (Prohance) | ic | 1^st^– 4^th^ ids feet & pp | T1w 3D GRE (VIBE) | Before & 15, 25, 35, 45 and 55 |
| Lohrmann^91^ | 1.5 | 1.8 | Gadodiamide (Omniscan) | ic | 1^st^–4^th^ ids feet & pp | T1w 3D GRE (VIBE) | 15 and 40 |
| Lohrmann^92^ | 1.5 | 1.8 | Gadodiamide (Omniscan) | ic | 1^st^–4^th^ ids feet & pp | T1w 3D GRE (VIBE) | Before & 15, 25, 35, 45 and 55 |
| Lohrmann^93^ | 1.5 | 1.8 | Gadodiamide (Omniscan) | ic | 1^st^–4^th^ ids feet & pp | T1w 3D GRE (VIBE) | Before & 5, 15, 25, 35, 45 and 55 |
| Lohrmann^94^ | 1.5 | 0.9 | Gadodiamide (Omniscan) | ic | 1^st^–4^th^ ids feet & pp | T1w 3D FLASH | Before & 5, 15, 25, 35, 45 and 55 |
| Yasunaga^95^ | 3.0 | 0.5 | Meglumine gadoterate (Magnescope) | sc | 1^st^ ids feet, medial and lateral malleoli, 5th metatarsal bone | T1w 3D GRE with FS | 10-25, 30-45 |
| Yasunga^96^ | 3.0 | 0.5 | Meglumine gadoterate (Magnescope) | sc | 1^st^–4^th^ ids feet, base of the 1st and 5th toe & medial and lateral malleoli | T1w 3D GRE with FS | 10-25, 30-45 |
| Pons^97^ | 3.0 | 0.8 | Gd-BOPTA (Multihance) | - | 2^nd^ and 4^th^ ids | T1w 3D GRE with FS | NR |
| Zeltzer^98^ | 3.0 | 0.8 | Gd-BOPTA (Multihance) | ic | 1^st^–4^th^ ids hand | T1w 3D GRE with FS | 15, 30 |
| Mazzei^99^ | 1.5 | 0.9 | Gd-BOPTA (Multihance) | sc | 1^st^ – 4^th^ ids hands or feet | T1w 3D GRE with FS | Before & 5, 20, 35 |
| Bae^100^ | 3.0 | 0.9 | Gadobutrol | ic | 1^st^ – 4^th^ ids hand | T1w 3D GRE with FS | Every 5 min for 70 min |
| Weiss^101^ | 3.0 | 1.0 | Gd-DPTA (Mangevist) | ic | 1^st^ – 3^rd^ ids feet | T1w 3D GRE with FS | 10, 20, 30, 40 |
| Notohamiprodjo^102^ | 3.0 | 0.6 | Gd-DPTA (Mangevist) | ic | 1^st^ – 3^rd^ ids feet | T1w 3D GRE with FS | NR |
| Kim^103^ | 1.5 | - | - | - | - | T2w STIR | - |
| Cellina^104^ | 1.5 | - | - | - | - | T2w 3D TSE | - |
| Cellina^105^ | 1.5 | - | - | - | - | T2w 3D FSE | - |
| Cresenzi^106^ | 3.0 | - | - | - | - | 3D TSE with SPIR FS | - |
| Liu^107^ | 1.5 | - | - | - | - | T2w 3D TSE with FS (HASTE) | - |
| Hou^108^ | NR | 0.5 | ^68^Ga-NOTA Evans Blue | sc | 1^st^ ids feet | TOF PET imaging  T1W FSE with FS | 20, 40 |
| Long^109^ | -  3.0 | 0.5  0.6 - 0.8 | ^68^Ga-NOTA Evans Blue  Gd-DTPA (Magnevist) | sc  sc | 1^st^ ids feet | PET  T1w 3D | 60, 90  NR |

*sc: subcutaneous; ic: intracutaneous; iv: intravenous; Gd-BOPTA: Gadobenate dimeglumine. Gd-DPTA: Gadopentate dimeglumine; VIBE: volumetric interpolated breath hold examination*

*GRE: gradient-recalled echo; TSE: turbo spin echo; VISTA: volumetric isotropic turbo-spin echo acquisition; FLASH: fast low angle shot; FS: fat suppression; STIR: short tau inversion recoverty; NR: not reported*

Supplementary table 9: Characteristics of included studies on ultrasound

| Study information | | Patient characteristics | | | | LE type (No.) | | | Limbs (No.) | |
| --- | --- | --- | --- | --- | --- | --- | --- | --- | --- | --- |
| Year | Author | M | F | Age*  (years) | ISL stage (%) | P | S | H | UL | LL |
|  |  | (No.) | |  |  |  |  |  |  |  |
| Diagnosis | | | | | | | | | | |
| 2021a | Hara^110^ | 0 | 14 | 59.7 (48 - 84) | I: 32.1 IIa: 25.0  IIb: 28.6 III: 14.3 | - | 14 | - | - | 28 |
| 2021c | Hara^111^ | 17 | 18 | 30.9 (23 – 55) | - | - | - | 35 | - | 70 |
| 2020 | Hara^112^ | 0 | 31 | 62.0 (42 - 86) | I: 34.4 IIa: 13.1  IIb: 42.6 III: 9.9 | 1 | 30 | - | - | 61 |
| Pre-operative LVB planning | | | | | | | | | | |
| 2022 | Visconti^113^ | 17 | 59 | 60.8 ± 12.6 | IIb: 75 III: 25 | - | 76 | - | 47 | 29 |
| 2021 | Mohos^114^ | 16 | 40 | 54.7 ± 14.7 | I: 12.8 II: 61.5  III: 25.7 | 16 | 40 | - | 13 | 48 |
| 2021b | Hara^115^ | 0 | 17 | 61 (42 – 83) | I: 31.3 IIa: 18.7  IIb: 31.3. III: 18.7 | - | 17 | - | - | 32 |
| 2020 | Czedik-Eysenberg^116^ | 7 | 21 | - | I: 3.8 II: 76.9  III: 19.2 | 10 | 18 | - | - | 28 |
| 2020 | Bianchi^117^ | 7 | 19 | 57.6 (42 - 78) | - | - | 26 | - | 14 | 12 |
| 2019 | Hayashi^118^ | 5 | 25 | 55.7 (36 - 74) | - | - | 30 | - | 13 | 17 |
| 2018 | Mihara^119^ | 2 | 42 | 61.4 (40 - 83) | I: 25.0 IIa: 21.2  IIb: 45.0 III: 8.8 | 2 | 42 | - | 3 | 77 |
| 2018 | Hayashi^120^ | 2 | 53 | 50.7 (32 - 84) | II: 100 | - | 55 | - | - | 55 |
| 2016 | Hayashi^121^ | 4 | 22 | 40 (19 - 53) | - | - | - | 26 | - | 26 |

**Values are mean (range) or ± standard deviation; M: male; F: female; ISL: International Society of Lymphology; P: primary; S: secondary; H: healthy; UL: upper limbs; LL: lower limbs; LE: lymphedema; NR: not reported*

Supplementary table 10: Ultrasound imaging methods and performance

| Author | Imaging system | f  (MHz) | Vessel diameter*  (mm) | Depth*  (mm) | Sensitivity  (%) | Specificity  (%) | Accuracy (%) | Gold standard |
| --- | --- | --- | --- | --- | --- | --- | --- | --- |
|  | | | | | Diagnostic performance | | |  |
| Hara^110^ | Noblus EUP-L65 (Hitachi Medical Corp.) | 18 | - | - | 95.0 | 100 | 94.6 | NIRF-L |
| Hara^111^ | Aplio i900 (Canon Medical Systems Corp.) | 33 | Thigh supine: 0.154  Thigh upright: 0.150  LL supine: 0.160  LL sitting: 0.163  LL upright: 0.164 | - | - | - | - | - |
| Hara^112^ | Noblus EUP-L65 (Hitachi Medical Corp.) | 18 | Thigh supine: 0.43 ± 0.02  Thigh upright: 0.40 ± 0.02  LL supine: 0.68 ± 0.04  LL sitting 0.63 ± 0.04  LL upright 0.63 ± 0.04 | - | - | - | - | - |
|  | | | | | Lymphatic vessel detection performance | | |  |
| Visconti^113^ | Vevo MD (FUJIFILM VisualSonics) | 48,  70 | - | - | - | - | - | NIRF-L |
| Mohos^114^ | Aplio i800 (Canon Medical Systems Corp.) | 24 | >0.2 | <19 | - | - | - |  |
| Hara^115^ | Noblus EUP-L65 (Hitachi Medical Corp.) | 18 | CSH: 0.65 ± 0.35  CSW: 1.3 ± 0.41  Significant correlation between CSH, CSW and intraoperatively measured diameter | - | - | - | - | Intraoperative findings |
| Czedik-Eysenberg^116^ | Aplio i800 (Canon Medical Systems Corp.) | 24 | Leg: 1.15 ± 0.47 | UL: 13.2 ± 3.9  LL 8.9 ± 3.5 | - | 90.2 | - | Intraoperative findings |
| Bianchi^117^ | Vevo MD (FUJIFILM VisualSonics) | 48, 70 | Significant correlation between inner and outer diameter and wall thickness between US and histology findings | - | - | - | - | Histology |
| Hayashi^118^ | *UHFUS*: Vevo MD (FUJIFILM VisualSonics)  *CHFUS*: Prosound F75 (Hitachi Medical Corp.) | *1*8, 70 | *UHFUS*:  Arm: 0.336 ± 0.008  Leg: 0.417 ± 0.001  *CHFUS*:  Arm: 0.403 ± 0.002  Leg: 0.468 ± 0.003 | *UHFUS*: 4.6  *CHFUS*: 6.4 | *UHFUS*: 94.9  *CHFUS*: 66.3 | *UHFUS*: 98.3  *CHFUS*: 91.3 | - | NIRF-L |
| Mihara^119^ | Noblus EUP-L65 (Hitachi Medical Corp.) | 18 | - | - | T: 82.3  N: 66.7  E: 82.9  C: 85.7  S: 85.7 | 100 | T: 46.8  N: 0  E: 62.9  C: 50.0  S: 14.2 | Intraoperative findings |
| Hayashi^120^ | Noblus EUP-L65 (Hitachi Medical Corp.) | 18 | 0.66 ± 1.18 | Groin: (<5 - 15)  Thigh: (5 - 25)  Knee (5 - 25)  LL: (<5 - 15) | 88.2 | 92.7 | - | Intraoperative findings |
| Hayashi^121^ | Noblus EUP-L65 (Hitachi Medical Corp.) | 15 | - |  | 95.5 | 92.9 | - | NIRF-L |

**Values are mean ± standard deviation; frequency; LL: lower leg; UL: upper leg; NIRF-L: near infrared fluorescence lymphography; mm: millimeter; CSH: cross-sectional height; CSW: cross-sectional width;*

Supplementary table 11: Characteristics of included studies on photoacoustic imaging

| Study information | | Patient characteristics | | | | LE type (No.) | | | Limbs (No.) | |
| --- | --- | --- | --- | --- | --- | --- | --- | --- | --- | --- |
| Year | Author | M | F | Age*  (years) | ISL stage (%) | P | S | H | UL | LL |
|  |  | (No.) | |  |  |  |  |  |  |  |
| 2022 | Suzuki^122^ | - | 5 | 59.4 | II: 100 | - | 5 | - | - | 5 |
| 2022 | Suzuki^123^ | - | 1**7** | 61 ± 11 | I: 11.8 IIa: 76.5 IIb: 5.9 III: 5.9 | - | 17 | - | - | 17 |
| 2022 | Suzuki^124^ | 4 | 15 | 42.9 ± 12.8 | - | - | - | 19 | - | 19 |
| 2022 | Oh^125^ | 4 | 15 | 62.4 ± 13.3 | I: 15.8 II: 73.7 III: 10.5 | - | 19 | - | 3 | 16 |
| 2020 | Kajita^126^ | NR | | NR | NR | - | 30 | 20 | 50 | |
| 2020 | Suzuki^127^ | 3 | 12 | 42 ± 12 | - | - | - | 15 | - | 15 |

Supplementary table 12: Contrast agent administration and imaging methods for photoacoustic imaging

| Author | Imaging System | Wavelengths  (nm) | Dose (mL) | Contrast agent | Type | Site |
| --- | --- | --- | --- | --- | --- | --- |
| Suzuki^122^ | PAI-05 (Luxonus, Japan) | 797 & 835 | NR | 0.5% Diagniogreen  (Daiichi Pharmaceutical) | NR | NR |
| Suzuki^123^ | PAI-05 (Luxonus, Japan) | 797 & 835 | 0.2 | 0.5% Diagniogreen  (Daiichi Pharmaceutical) | sc | 1^st^ and 4^th^ ids feet & lateral malleolus |
| Suzuki^124^ | PAI-05 (Luxonus, Japan) | 797 & 835 | 0.2 | 0.5% Diagniogreen  (Daiichi Pharmaceutical) | sc | 1^st^ and 4^th^ ids feet & lateral malleolus |
| Oh^125^ | PAI-05 (Luxonus, Japan) | 797 & 835 | 0.1 | 0.5% ICG | sc | 3 sites dorsal aspect hands |
| Kajita^126^ | PAI-05 (Luxonus, Japan) | 797 & 835 | NR | NR | sc | Dorsal aspect of each foot or hands |
| Suzuki^127^ | PAI-05 (Luxonus, Japan) | 797 & 835 | 0.2 | 0.5% Diagniogreen  (Daiichi Pharmaceutical) | sc | 1^st^ and 4^th^ ids feet & lateral malleolus |

# References

1. Bourgeois P, et al. Edemas of the face and lymphoscintigraphic examination. *Sci rep*. 2021;11(1):6444. doi:10.1038/s41598-021-85835-w

2. Pappalardo M, et al. Staging and clinical correlations of lymphoscintigraphy for unilateral gynecological cancer–related lymphedema. *Journal of Surgical Oncology*. 2020;121(3):422-434. doi:10.1002/jso.25817

3. Campisi CC, et al. Rationale for Study of the Deep Subfascial Lymphatic Vessels During Lymphoscintigraphy for the Diagnosis of Peripheral Lymphedema. *Clin Nucl Med*. 2019;44(2):91-98. doi:10.1097/rlu.0000000000002400

4. Tartaglione G, et al. Stress lymphoscintigraphy for early detection and management of secondary limb lymphedema. *Clin Nucl Med*. 2018;43(3):155-161. doi:10.1097/rlu.0000000000001963

5. Cheng MH, et al. Validity of the Novel Taiwan Lymphoscintigraphy Staging and Correlation of Cheng Lymphedema Grading for Unilateral Extremity Lymphedema. *Ann Surg*. 2018;268(3):513-525. doi:10.1097/sla.0000000000002917

6. Maclellan RA, et al. Correlation Between Lymphedema Disease Severity and Lymphoscintigraphic Findings: A Clinical-Radiologic Study. *J Am Coll Surg*. 2017;225(3):366-370. doi:10.1016/j.jamcollsurg.2017.06.005

7. Hassanein AH, et al. Diagnostic Accuracy of Lymphoscintigraphy for Lymphedema and Analysis of False-Negative Tests. *Plastic and Reconstructive Surgery-Global Open*. 2017;5(7):e1396. doi:10.1097/GOX.0000000000001396

8. Yoo JN, et al. Validity of Quantitative Lymphoscintigraphy as a Lymphedema Assessment Tool for Patients With Breast Cancer. *Annals of Rehabilitation Medicine-Arm*. 2015;39(6):931-940. doi:10.5535/arm.2015.39.6.931

9. Devoogdt N, et al. Reproducibility of lymphoscintigraphic evaluation of the upper limb. *Lymphatic Res Biol*. 2014;12(3):175-184. doi:10.1089/lrb.2013.0034

10. Kalawat TC, et al. Role of lymphoscintigraphy in diagnosis and management of patients with leg swelling of unclear etiology. *Indian J Nucl Med*. 2012;27(4):226-230. doi:10.4103/0972-3919.115392

11. Infante JR, et al. Lymphoscintigraphy for differential diagnosis of peripheral edema: Diagnostic yield of different scintigraphic patterns. *Rev Esp Med Nucl Imagen Mol*. 2012;31(5):237-242. doi:10.1016/j.remn.2011.11.011

12. Mikami T, et al. Classification of lymphoscintigraphy and relevance to surgical indication for lymphaticovenous anastomosis in upper limb lymphedema. *Lymphology*. 2011;44(4):155-167.

13. Maegawa J, et al. Types of lymphoscintigraphy and indications for lymphaticovenous anastomosis. *Microsurgery*. 2010;30(6):437-442. doi:10.1002/micr.20772

14. Pecking AP, et al. Relationship between lymphoscintigraphy and clinical findings in lower limb lymphedema (LO): Toward a comprehensive staging. *Lymphology*. 2008;41(1):1-10.

15. Dabrowski J, et al. Optimized lymphoscintigraphy and diagnostics of lymphatic oedema of the lower extremities. *Nucl Med Rev*. 2008;11(1):26-29.

16. Williams WH, et al. Radionuclide lymphangioscintigraphy in the evaluation of peripheral lymphedema. *Clin Nucl Med*. 2000;25(6):451-464. doi:10.1097/00003072-200006000-00013

17. Kwon HR, et al. Predictive role of lymphoscintigraphy undergoing lymphovenous anastomosis in patients with lower extremity lymphedema: a preliminary study. *BMC Med Imaging*. 2021;21(1):188. doi:10.1186/s12880-021-00713-1

18. Kim HO, et al. Lymphoscintigraphic Findings as Indicators of Lymphaticovenous Anastomosis Outcome in Patients with Extremity Lymphedema: A Retrospective Cohort Study. *Clin Nucl Med*. 2021;46(7):549-555. doi:10.1097/rlu.0000000000003630

19. Kim YH, et al. Predictive value of lymphoscintigraphy in patients with breast cancer-related lymphedema undergoing complex decongestive therapy. *Breast Cancer Res Treat*. 2019;173(3):735-741. doi:10.1007/s10549-018-5041-2

20. Chiewvit S, Kumnerdnakta S. Lymphoscintigraphic findings that predict favorable outcome after lymphaticovenous anastomosis. *Lymphology*. 2017;50(1):1-8.

21. Bourgeois P, Leduc O. Value of one additional injection at the root of the limb in the lymphoscintigraphic evaluation and management of primary and secondary lower-limb lymphedemas. *PLoS ONE*. 2021;16(7):e0253900. doi:10.1371/journal.pone.0253900

22. Tartaglione G, et al. Intradermal lymphoscintigraphy at rest and after exercise: A new technique for the functional assessment of the lymphatic system in patients with lymphoedema. *Nucl Med Commun*. 2010;31(6):547-551. doi:10.1097/MNM.0b013e328338277d

23. O'Mahony S, et al. Imaging of lymphatic vessels in breast cancer-related lymphedema: Intradermal versus subcutaneous injection of 99mTc-immunoglobulin. *Am J Roentgenol*. 2006;186(5):1349-1355. doi:10.2214/ajr.04.1341

24. O'Mahony S, et al. Finding an optimal method for imaging lymphatic vessels of the upper limb. *Eur J Nucl Med Mol Imaging*. 2004;31(4):555-563. doi:10.1007/s00259-003-1399-3

25. Stanton AW, et al. Impairment of lymph drainage in subfascial compartment of forearm in breast cancer-related lymphedema. *Lymphat Res Biol*. 2003;1(2):121-132. doi:10.1089/153968503321642615

26. Fujiyoshi T, et al. Pathological Changes in the Lymphatic System of Patients with Secondary Lower Limb Lymphedema Based on Single Photon-Emission Computed Tomography/Computed Tomography/Lymphoscintigraphy Images. *Lymphat Res Biol*. 2021;20(2):144-152. doi:10.1089/lrb.2021.0040

27. Baulieu F, et al. Lymphoscintigraphy in limb lymphoedema: Current methodology and interests. *Med Nucl*. 2015;39(1):26-42. doi:10.1016/j.mednuc.2015.02.009

28. Baulieu F, et al. Contributions of SPECT/CT imaging to the lymphoscintigraphy investigations of the lower limb lymphedema. *Lymphology*. 2013;46(3):106-119.

29. Pecking AP, et al. SPECT-CT fusion imaging radionuclide lymphoscintigraphy: potential for limb lymphedema assessment and sentinel node detection in breast cancer. *Cancer Treat Res*. 2007;135:79-84. doi:10.1007/978-0-387-69219-7_6

30. Pappalardo M, et al. Staging and clinical correlations of lymphoscintigraphy for unilateral gynecological cancer-related lymphedema. *J Surg Oncol*. 2020;121(3):422-434. doi:10.1002/jso.25817

31. Tokumoto H, et al. Relationship Between the Circumference Difference and Findings of Indocyanine Green Lymphography in Breast Cancer-Related Lymphedema. *Ann Plast Surg*. 2022;88(1):114-117. doi:10.1097/sap.0000000000002918

32. Thomis S, et al. Relation Between Early Disturbance of lymphatic transport Visualized With Lymphofluoroscopy and Other Clinical Assessment Methods in Patients With Breast Cancer. *Clin Breast Cancer*. 2022;22(1):e37-e47. doi:10.1016/j.clbc.2021.06.015

33. Thomis S, et al. The Interrater Reliability of the Scoring of the Lymphatic Architecture and Transport Through Near-InfraRed Fluorescence Lymphatic Imaging in Patients with Breast Cancer-Related Lymphedema. *Lymphat Res Biol*. 2021;20(2):133-143.

34. Jørgensen MG, et al. Indocyanine green lymphangiography is superior to clinical staging in breast cancer-related lymphedema. *Sci Rep*. 2021;11(1):21103. doi:10.1038/s41598-021-00396-2

35. Akita S, et al. A phase III, multicenter, single-arm study to assess the utility of indocyanine green fluorescent lymphography in the treatment of secondary lymphedema. *J Vasc Surg Venous Lymphatic Disord*. 2021;10(3):728-737.e3. doi:10.1016/j.jvsv.2021.09.006

36. Jørgensen MG, et al. Prospective validation of indocyanine green lymphangiography staging of breast cancer-related lymphedema. *Cancers*. 2021;13(7):1540. doi:10.3390/cancers13071540

37. Thomis S, et al. Correlation between Clinical Assessment and Lymphofluoroscopy in Patients with Breast Cancer-Related Lymphedema: A Study of Concurrent Validity. *Lymphatic Res Biol*. 2020;18(6):539-548. doi:10.1089/lrb.2019.0090

38. Medina-Rodríguez ME, et al. Relationship between perimetric increase and fluoroscopic pattern type in secondary upper limb lymphedema observed by Indocyanine green lymphography. *Medicine (Baltimore)*. 2020;99(24):e20432. doi:10.1097/md.0000000000020432

39. Kinugawa K, et al. Lymph Vessel Mapping Using Indocyanine Green Lymphography in the Nonaffected Side of Lower Leg. *Plast reconstr surg, Glob open*. 2020;8(6):e2929. doi:10.1097/GOX.0000000000002929

40. Lee YW, et al. Lymphatic vessel mapping in the upper extremities of a healthy Korean population. *Arch Plast Surg*. 2018;45(2):152-157. doi:10.5999/aps.2017.00983

41. Suami H, et al. A new indocyanine green fluorescence lymphography protocol for identification of the lymphatic drainage pathway for patients with breast cancer-related lymphoedema. *BMC Cancer*. 2019;19(1):985. doi:10.1186/s12885-019-6192-1

42. Matsumoto K, et al. Exercise-Loaded Indocyanine Green Fluorescence Lymphangiography for Diagnosing Lymphedema. *J Reconstr Microsurg*. 2019;35(2):138-144. doi:10.1055/s-0038-1667366

43. Garza RM, et al. The Relationship Between Clinical and Indocyanine Green Staging in Lymphedema. *Lymphat res biol*. 2019;17(3):329-333. doi:10.1089/lrb.2018.0014

44. Shinaoka A, et al. Accelerated Lymph Flow in Early-Stage Secondary Lymphedema Detected by Indocyanine Green Fluorescence Lymphography. *J Reconstr Microsurg*. 2017;33(8):596-602. doi:10.1055/s-0037-1603740

45. Gentileschi S, et al. Lymphatic mapping of the upper limb with lymphedema before lymphatic supermicrosurgery by mirroring of the healthy limb. *Microsurgery*. 2017;37(8):881-889. doi:10.1002/micr.30247

46. Tashiro K, et al. Proximal and distal patterns: Different spreading patterns of indocyanine green lymphography in secondary lower extremity lymphedema. *J Plast Reconstr Aesthetic Surg*. 2016;69(3):368-375. doi:10.1016/j.bjps.2015.10.042

47. Akita S, et al. Early Detection of Lymphatic Disorder and Treatment for Lymphedema following Breast Cancer. *Plast Reconstr Surg*. 2016;138(2):192e-202e. doi:10.1097/prs.0000000000002337

48. Mihara M, et al. Predictive lymphatic mapping: a method for mapping lymphatic channels in patients with advanced unilateral lymphedema using indocyanine green lymphography. *Ann Plast Surg*. 2014;72(6):706-710. doi:10.1097/SAP.0b013e31826a18b1

49. Mihara M, et al. High-accuracy diagnosis and regional classification of lymphedema using indocyanine green fluorescent lymphography after gynecologic cancer treatment. *Ann Plast Surg*. 2014;72(2):204-208. doi:10.1097/SAP.0b013e3182586b79

50. Akita S, et al. Early diagnosis and risk factors for lymphedema following lymph node dissection for gynecologic cancer. *Plast Reconstr Surg*. 2013;131(2):283-290. doi:10.1097/PRS.0b013e318277870f

51. Suami H, et al. Using indocyanine green fluorescent lymphography to demonstrate lymphatic architecture. *J Lymphoedema*. 2012;7(2):25-29.

52. Aldrich MB, et al. Lymphatic abnormalities in the normal contralateral arms of subjects with breast cancer-related lymphedema as assessed by near-infrared fluorescent imaging. *Biomed Opt Express*. 2012;3(6):1256-1265. doi:10.1364/BOE.3.001256

53. Yamamoto T, et al. The earliest finding of indocyanine green lymphography in asymptomatic limbs of lower extremity lymphedema patients secondary to cancer treatment: the modified dermal backflow stage and concept of subclinical lymphedema. *Plast Reconstr Surg*. 2011;128(4):314e-321e. doi:10.1097/PRS.0b013e3182268da8

54. Yamamoto T, et al. Indocyanine green-enhanced lymphography for upper extremity lymphedema: a novel severity staging system using dermal backflow patterns. *Plast Reconstr Surg*. 2011;128(4):941-947. doi:10.1097/PRS.0b013e3182268cd9

55. Yamamoto T, et al. Characteristic indocyanine green lymphography findings in lower extremity lymphedema: the generation of a novel lymphedema severity staging system using dermal backflow patterns. *Plast Reconstr Surg*. 2011;127(5):1979-1986. doi:10.1097/PRS.0b013e31820cf5df

56. Unno N, et al. Preliminary experience with a novel fluorescence lymphography using indocyanine green in patients with secondary lymphedema. *J Vasc Surg*. 2007;45(5):1016-1021. doi:10.1016/j.jvs.2007.01.023

57. Johnson AR, et al. Real-time visualization of the mascagni-sappey pathway utilizing ICG lymphography. *Cancers*. 2020;12(5):1195. doi:10.3390/cancers12051195

58. Tashiro K, et al. Visualization of Accessory Lymphatic Pathways in Secondary Upper Extremity Lymphedema Using Indocyanine Green Lymphography. *Ann Plast Surg*. 2017;79(4):393-396. doi:10.1097/sap.0000000000001120

59. Kelly B, et al. Function of Upper Extremity Human Lymphatics Assessed by Near-Infrared Fluorescence Imaging. *Lymphat Res Biol*. 2020;18(3):226-231. doi:10.1089/lrb.2019.0041

60. Granoff MD, et al. A Novel Approach to Quantifying Lymphatic Contractility during Indocyanine Green Lymphangiography. *Plast Reconstr Surg*. 2019;144(5):1197-1201. doi:10.1097/prs.0000000000006176

61. Groenlund JH, et al. A Validation Study of Near-Infrared Fluorescence Imaging of Lymphatic Vessels in Humans. *Lymphat Res Biol*. 2017;15(3):227-234. doi:10.1089/lrb.2016.0061

62. Yamamoto T, et al. Dynamic Indocyanine Green (ICG) lymphography for breast cancer-related arm lymphedema. *Ann Plast Surg*. 2014;73(6):706-709. doi:10.1097/SAP.0b013e318285875f

63. Yamamoto T, et al. Indocyanine green velocity: lymph transportation capacity deterioration with progression of lymphedema. *Ann Plast Surg*. 2013;71(5):591-594. doi:10.1097/SAP.0b013e318255168a

64. Rasmussen JC, et al. Human lymphatic architecture and dynamic transport imaged using near-infrared fluorescence. *Transl Oncol*. 2010;3(6):362-372. doi:10.1593/tlo.10190

65. Unno N, et al. Quantitative lymph imaging for assessment of lymph function using indocyanine green fluorescence lymphography. *Eur J Vasc Endovasc Surg*. 2008;36(2):230-236. doi:10.1016/j.ejvs.2008.04.013

66. Shinaoka A, et al. A new severity classification of lower limb secondary lymphedema based on lymphatic pathway defects in an indocyanine green fluorescent lymphography study. *Sci Rep*. 2022;12(1):309. doi:10.1038/s41598-021-03637-6

67. Hara H, Mihara M. Classification of the lymphatic pathways in each lymphosome based on multi-lymphosome indocyanine green lymphography: Saphenous, calf, and thigh (SCaT) classification. *J Plast Reconstr Aesthetic Surg*. 2021;74(11):2941-2946. doi:10.1016/j.bjps.2021.03.078

68. Hara H, Mihara M. Multilymphosome injection indocyanine green lymphography can detect more lymphatic vessels than lymphoscintigraphy in lymphedematous limbs. *J Plast Reconstr Aesthetic Surg*. 2020;73(6):1025-1030. doi:10.1016/j.bjps.2020.01.021

69. Hara H, Mihara M. Multi-area lymphaticovenous anastomosis with multi-lymphosome injection in indocyanine green lymphography: A prospective study. *Microsurgery*. 2019;39(2):167-173. doi:10.1002/micr.30398

70. Hara H, et al. Comparison of indocyanine green lymphographic findings with the conditions of collecting lymphatic vessels of limbs in patients with lymphedema. *Plast Reconstr Surg*. 2013;132(6):1612-1618. doi:10.1097/PRS.0b013e3182a97edc

71. Yoon JA, et al. Indocyanine green lymphography and lymphoscintigraphy severity stage showed strong correlation in lower limb lymphedema. *Lymphatic Res Biol*. 2021;19(1):80-85. doi:10.1089/lrb.2020.0043

72. Yoon JA, et al. Correlation of ICG lymphography and lymphoscintigraphy severity stage in secondary upper limb lymphedema. *J Plast Reconstr Aesthetic Surg*. 2020;73(11):1982-1988. doi:10.1016/j.bjps.2020.08.055

73. Mihara M, et al. Indocyanine green lymphography is superior to lymphoscintigraphy in imaging diagnosis of secondary lymphedema of the lower limbs. *J Vasc Surg Venous Lymphatic Disord*. 2013;1(2):194-201. doi:10.1016/j.jvsv.2012.07.011

74. Akita S, et al. Comparison of lymphoscintigraphy and indocyanine green lymphography for the diagnosis of extremity lymphoedema. *J Plast Reconstr Aesthetic Surg*. 2013;66(6):792-798. doi:10.1016/j.bjps.2013.02.023

75. Soga S, et al. Lower limb lymphedema staging based on magnetic resonance lymphangiography. *J Vasc Surg Venous Lymphatic Disord*. 2022;10(2):445-453.e3. doi:10.1016/j.jvsv.2021.06.006

76. Wolfs JAGN, et al. Visualization of Both the Superficial and Deep Lymphatic System of the Upper Extremity Using Magnetic Resonance Lymphography. *Lymphat Res Biol*. 2021, 10.1089/lrb.2021.0012. doi:10.1089/lrb.2021.0012

77. Soga S, et al. Analysis of collateral lymphatic circulation in patients with lower limb lymphedema using magnetic resonance lymphangiography. *J Vasc Surg Venous Lymphatic Disord*. 2021;9(2):471-481.e1. doi:10.1016/j.jvsv.2020.04.029

78. Sheng L, et al. Magnetic Resonance Lymphography of Lymphatic Vessels in Upper Extremity With Breast Cancer-Related Lymphedema. *Ann Plast Surg*. 2020;84(1):100-105. doi:10.1097/sap.0000000000001994

79. Abdelfattah U, et al. Correlation between superficial and deep lymphatic systems using magnetic resonance lymphangiography in breast cancer-related lymphedema: Clinical implications. *J Plast Reconstr Aesthetic Surg*. 2020;73(6):1018-1024. doi:10.1016/j.bjps.2019.11.053

80. Ripley B, et al. Initial clinical experience with dual-agent relaxation contrast for isolated lymphatic channel mapping. *Radiology*. 2018;286(2):705-714. doi:10.1148/radiol.2017170241

81. Gennaro P, et al. Could MRI visualize the invisible? An Italian single center study comparing magnetic resonance lymphography (MRL), super microsurgery and histology in the identification of lymphatic vessels. *Eur Rev Med Pharmacol Sci*. 2017;21(4):687-694.

82. Jeon JY, et al. Three-dimensional isotropic fast spin-echo MR lymphangiography of T1-weighted and intermediate-weighted pulse sequences in patients with lymphoedema. *Clin Radiol*. 2016;71(1):e56-e63. doi:10.1016/j.crad.2015.10.015

83. Liu N, Zhang Y. Magnetic Resonance Lymphangiography for the Study of Lymphatic System in Lymphedema. *J Reconstr Microsurg*. 2016;32(1):66-71. doi:10.1055/s-0034-1384213

84. Borri M, et al. Quantitative Contrast-Enhanced Magnetic Resonance Lymphangiography of the Upper Limbs in Breast Cancer Related Lymphedema: An Exploratory Study. *Lymphatic Res Biol*. 2015;13(2):100-106. doi:10.1089/lrb.2014.0039

85. Zhou GX, et al. MR lymphangiography at 3.0 Tesla to assess the function of inguinal lymph node in low extremity lymphedema. *J Magn Reson Imaging*. 2014;40(6):1430-1436. doi:10.1002/jmri.24499

86. Liu NF, et al. Magnetic resonance lymphography demonstrates spontaneous lymphatic disruption and regeneration in obstructive lymphedema. *Lymphology*. 2013;46(2):56-63.

87. Lu Q, et al. MR Lymphography of Lymphatic Vessels in Lower Extremity with Gynecologic Oncology-Related Lymphedema. *PLoS ONE*. 2012;7(11):e50319. doi:10.1371/journal.pone.0050319

88. Notohamiprodjo M, et al. MR-lymphangiography at 3.0T - A feasibility study. *Eur Radiol*. 2009;19(11):2771-2778. doi:10.1007/s00330-009-1461-z

89. Dimakakos EP, et al. Interstitial magnetic resonance lymphography: The clinical effectiveness of a new method. *Lymphology*. 2008;41(3):116-125.

90. Lohrmann C, et al. Gadoteridol for MR imaging of lymphatic vessels in lymphoedematous patients: Initial experience after intracutaneous injection. *Br J Radiol*. 2007;80(955):569-573. doi:10.1259/bjr/95414884

91. Lohrmann C, et al. Magnetic resonance imaging of lymphatic vessels without image subtraction: A practicable imaging method for routine clinical practice? *J Comput Assisted Tomogr*. 2007;31(2):303-308. doi:10.1097/01.rct.0000237814.33925.32

92. Lohrmann C, et al. MR imaging of the lymphatic system: Distribution and contrast enhancement of gadodiamide after intradermal injection. *Lymphology*. 2006;39(4):156-163.

93. Lohrmann C, et al. High-resolution MR lymphangiography in patients with primary and secondary lymphedema. *American Journal of Roentgenology*. 2006;187(2):556-561. doi:10.2214/AJR.05.1750

94. Lohrmann C, et al. Indirect magnetic resonance lymphangiography in patients with lymphedema. Preliminary results in humans. *Eur J Radiol*. 2006;59(3):401-406. doi:10.1016/j.ejrad.2006.02.012

95. Yasunaga Y, et al. Impact of Magnetic Resonance Lymphography on Lymphaticolvenular Anastomosis for Lower-Limb Lymphedema. *J Reconstr Microsurg*. 2022;38(2):121-128. doi:10.1055/s-0041-1731638

96. Yasunaga Y, et al. Magnetic resonance lymphography as three-dimensional navigation for lymphaticovenular anastomosis in patients with leg lymphedema. *J Plast Reconstr Aesthetic Surg*. 2020;74(6):1253-1260. doi:10.1016/j.bjps.2020.10.099

97. Pons G, et al. Preoperative planning of lymphaticovenous anastomosis: The use of magnetic resonance lymphangiography as a complement to indocyanine green lymphography. *J Plast Reconstr Aesthet Surg*. 2019;72(6):884-891. doi:10.1016/j.bjps.2019.02.024

98. Zeltzer AA, et al. MR lymphography in patients with upper limb lymphedema: The GPS for feasibility and surgical planning for lympho-venous bypass. *J Surg Oncol*. 2018;118(3):407-415. doi:10.1002/jso.25145

99. Mazzei MA, et al. High-resolution MR lymphangiography for planning lymphaticovenous anastomosis treatment: a single-centre experience. *Radiol Med*. 2017;122(12):918-927. doi:10.1007/s11547-017-0795-x

100. Bae JS, et al. Evaluation of lymphedema in upper extremities by MR lymphangiography: Comparison with lymphoscintigraphy. *Magn Reson Imaging*. 2018;49:63-70. doi:10.1016/j.mri.2017.12.024

101. Weiss M, et al. Magnetic resonance imaging versus lymphoscintigraphy for the assessment of focal lymphatic transport disorders of the lower limb: First experiences. *NuklearMedizin*. 2014;53(5):190-196. doi:10.3413/Nukmed-0649-14-03

102. Notohamiprodjo M, et al. MR lymphangiography at 3.0 T: Correlation with lymphoscintigraphy. *Radiology*. 2012;264(1):78-87. doi:10.1148/radiol.12110229

103. Kim G, et al. Use of non-contrast MR in diagnosing secondary lymphedema of the upper extremities. *Clin Imaging*. 2021;80:400-405. doi:10.1016/j.clinimag.2021.08.018

104. Cellina M, et al. Noncontrast MR Lymphography in Secondary Lower Limb Lymphedema. *J Magn Reson Imaging*. 2020;53(2):458-466. doi:10.1002/jmri.27328

105. Cellina M, et al. Noncontrast Magnetic Resonance Lymphography in Secondary Lymphedema Due to Prostate Cancer. *Lymphat Res Biol*. 2020;126(11):1477-1486. doi:10.1089/lrb.2020.0034

106. Crescenzi R, et al. Lymphedema evaluation using noninvasive 3T MR lymphangiography. *J Magn Reson Imaging*. 2017;46(5):1349-1360. doi:10.1002/jmri.25670

107. Liu N, et al. Noncontrast three-dimensional magnetic resonance imaging vs lymphoscintigraphy in the evaluation of lymph circulation disorders: A comparative study. *J Vasc Surg*. 2005;41(1):65-75. doi:10.1016/j.jvs.2004.11.013

108. Hou G, et al. 68Ga-NOTA-Evans Blue TOF PET/MR Lymphoscintigraphy Evaluation of the Severity of Lower Limb Lymphedema. *Clin Nucl Med*. 2019;44(6):439-445. doi:10.1097/rlu.0000000000002584

109. Long X, et al. Microsurgery guided by sequential preoperative lymphography using 68Ga-NEB PET and MRI in patients with lower-limb lymphedema. *Eur J Nucl Med Mol Imaging*. 2017;44(9):1501-1510. doi:10.1007/s00259-017-3676-6

110. Hara H, Mihara M. Diagnosis of Lymphatic Dysfunction by Evaluation of Lymphatic Degeneration with Lymphatic Ultrasound. *Lymphat Res Biol*. 2021;19(4):334-339. doi:10.1089/lrb.2019.0071

111. Hara H, Mihara M. Evaluation of lymphatic vessel diameters in healthy people using lymphatic ultrasound examination. *J Vasc Surg Venous Lymphatic Disord*. 2021;10(3):758-764. doi:10.1016/j.jvsv.2021.09.007

112. Hara H, Mihara M. Change of the Lymphatic Diameter in Different Body Positions. *Lymphat Res Biol*. 2020;19(3):249-255. doi:10.1089/lrb.2020.0081

113. Visconti G, et al. Lymphaticovenular Anastomosis for Advanced-Stage Peripheral Lymphedema: Expanding Indication and Introducing the Hand/Foot Sign. *Journal of Plastic, Reconstructive and Aesthetic Surgery*. 2022, 10.1016/j.bjps.2022.02.012. doi:10.1016/j.bjps.2022.02.012

114. Mohos B, et al. Long-term Use of Ultrasound for Locating Optimal LVA Sites: A Descriptive Data Analysis. *J Reconstr Microsurg*. 2022;38(3):238-244. doi:10.1055/s-0041-1740124

115. Hara H, Mihara M. The accuracy of lymphatic ultrasound in measuring the lymphatic vessel size in lower limb lymphedema patients. *J Plast Reconstr Aesthetic Surg*. 2021;S1748-6815(21):00651-3. doi:10.1016/j.bjps.2021.11.104

116. Czedik-Eysenberg M, et al. Exclusive use of ultrasound for locating optimal LVA sites—A descriptive data analysis. *J Surg Oncol*. 2020;121(1):51-56. doi:10.1002/jso.25728

117. Bianchi A, et al. Ultra-High frequency ultrasound imaging of lymphatic channels correlates with their histological features: A step forward in lymphatic surgery. *J Plast Reconstr Aesthetic Surg*. 2020;73(9):1622-1629. doi:10.1016/j.bjps.2020.05.053

118. Hayashi A, et al. Ultra High-frequency Ultrasonographic Imaging with 70 MHz Scanner for Visualization of the Lymphatic Vessels. *Plast reconstr surg, Glob open*. 2019;7(1):e2086. doi:10.1097/GOX.0000000000002086

119. Mihara M, et al. Ultrasonography for classifying lymphatic sclerosis types and deciding optimal sites for lymphatic-venous anastomosis in patients with lymphoedema. *Journal of Plastic Reconstructive and Aesthetic Surgery*. 2018;71(9):1274-1281. doi:10.1016/j.bjps.2018.05.012

120. Hayashi A, et al. Effective and efficient lymphaticovenular anastomosis using preoperative ultrasound detection technique of lymphatic vessels in lower extremity lymphedema. *J Surg Oncol*. 2018;117(2):290-298. doi:10.1002/jso.24812

121. Hayashi A, et al. Ultrasound visualization of the lymphatic vessels in the lower leg. *Microsurgery*. 2016;36(5):397-401. doi:10.1002/micr.22414

122. Suzuki Y, et al. Surgical applications of lymphatic vessel visualization using photoacoustic imaging and augmented reality. *J Clin Med*. 2022;11(1):194. doi:10.3390/jcm11010194

123. Suzuki Y, et al. Photoacoustic lymphangiography exhibits advantages over near-infrared fluorescence lymphangiography as a diagnostic tool in patients with lymphedema. *J Vasc Surg Venous Lymphatic Disord*. 2022;10(2):454-462.e1. doi:10.1016/j.jvsv.2021.07.012

124. Suzuki Y, et al. Use of photoacoustic imaging to determine the effects of aging on lower extremity lymphatic vessel function. *J Vasc Surg Venous Lymphatic Disord*. 2022;10(1):125-130. doi:10.1016/j.jvsv.2021.05.001

125. Oh A, et al. Three-dimensional analysis of dermal backflow in cancer-related lymphedema using photoacoustic lymphangiography. *Arch plast surg*. 2022;49(1):99-107. doi:10.5999/aps.2021.01235

126. Kajita H, et al. Photoacoustic lymphangiography. *Journal of Surgical Oncology*. 2020;121(1):48-50. doi:10.1002/jso.25575

127. Suzuki Y, et al. Subcutaneous lymphatic vessels in the lower extremities: Comparison between photoacoustic lymphangiography and near-infrared fluorescence lymphangiography. *Radiology*. 2020;295(2):469-474. doi:10.1148/radiol.2020191710
